# Repeated turnovers keep sex chromosomes young in willows

**DOI:** 10.1186/s13059-022-02769-w

**Published:** 2022-09-23

**Authors:** Deyan Wang, Yiling Li, Mengmeng Li, Wenlu Yang, Xinzhi Ma, Lei Zhang, Yubo Wang, Yanlin Feng, Yuanyuan Zhang, Ran Zhou, Brian J. Sanderson, Ken Keefover-Ring, Tongming Yin, Lawrence B. Smart, Stephen P. DiFazio, Jianquan Liu, Matthew Olson, Tao Ma

**Affiliations:** 1 Key Laboratory for Bio-Resource and Eco-Environment of Ministry of Education & Sichuan Zoige Alpine Wetland Ecosystem National Observation and Research Station, College of Life Science, Sichuan University, Chengdu, China; 2 Department of Biology, West Virginia University, Morgantown, WV USA; 3 Department of Biological Sciences, Texas Tech University, Lubbock, TX USA; 4 Departments of Botany and Geography, University of Wisconsin-Madison, Madison, WI USA; 5 The Key Laboratory of Tree Genetics and Biotechnology of Jiangsu Province and Education Department of China, Nanjing Forestry University, Nanjing, China; 6 Horticulture Section, School of Integrative Plant Science, Cornell University, Cornell AgriTech, Geneva, NY USA

**Keywords:** *Salix*, Genome, Sex determination, Sex chromosome turnover, Deleterious mutation load

## Abstract

**Background:**

Salicaceae species have diverse sex determination systems and frequent sex chromosome turnovers. However, compared with poplars, the diversity of sex determination in willows is poorly understood, and little is known about the evolutionary forces driving their turnover. Here, we characterized the sex determination in two *Salix* species, *S. chaenomeloides* and *S. arbutifolia*, which have an XY system on chromosome 7 and 15, respectively.

**Results:**

Based on the assemblies of their sex determination regions, we found that the sex determination mechanism of willows may have underlying similarities with poplars, both involving intact and/or partial homologs of a type A cytokinin response regulator (*RR*) gene. Comparative analyses suggested that at least two sex turnover events have occurred in *Salix*, one preserving the ancestral pattern of male heterogamety, and the other changing heterogametic sex from XY to ZW, which could be partly explained by the “deleterious mutation load” and “sexually antagonistic selection” theoretical models. We hypothesize that these repeated turnovers keep sex chromosomes of willow species in a perpetually young state, leading to limited degeneration.

**Conclusions:**

Our findings further improve the evolutionary trajectory of sex chromosomes in Salicaceae species, explore the evolutionary forces driving the repeated turnovers of their sex chromosomes, and provide a valuable reference for the study of sex chromosomes in other species.

**Supplementary Information:**

The online version contains supplementary material available at 10.1186/s13059-022-02769-w.

## Background

Sex chromosomes have arisen many times in diverse lineages [1], but the prevalence of inversions that lead to recombination suppression, the patterns of diversity related to early sex chromosome divergence, and the drivers of the early stages of sex chromosome evolution remain understudied. These early stages are often characterized by small regions that lack recombination between the X and Y (or Z & W) chromosomes that may expand over time [2–5], sometimes leading to the formation of “evolutionary strata” when expansions are episodic [6–8]. At the same time, the suppression of recombination is expected to lead to degeneration of the non-recombining Y or W chromosome resulting from gene loss and the accumulation of deleterious mutations and repeat elements caused by Muller’s Ratchet [9] and Hill-Robertson effects [10]. Ultimately, these processes are believed to be responsible for the formation of heteromorphic sex chromosomes that differ in size, morphology, and gene content, such as the sex chromosomes of mammals, birds, and some plants like the white campion [11–16].

Homomorphic sex chromosomes, which do not differ in size at the karyotype level and usually have a relatively small non-recombining region, have been identified in several animals such as fish, frogs, and lizards [17–19], as well as in many plants including kiwifruit, asparagus, and persimmons [3, 20–23]. Recent studies have uncovered high diversity in the genomic locations and sizes of the non-recombining regions in several taxa such as cichlid fishes, true frogs, and the plant families Rosaceae and Salicaceae [5, 24–30]. These results have inspired the hypotheses that rapid turnover of sex chromosomes, rare recombination highlighted by the “fountain-of-youth” hypothesis [31], or both result in the absence of degeneration and the lack of differentiation [32–34]. Multiple theoretical models have explored the evolutionary forces that drive the turnover of sex determination loci [35], but two hypotheses have garnered the majority of attention. First, strong selection on sexually antagonistic alleles may result in the turnover or movement of the sex chromosome [24, 36, 37]. Second, the “hot potato” model posits that the accumulation of deleterious mutation load on the non-recombining regions of the sex chromosomes is expected to favor a shift in the control of sex determination to new genomic regions that are unlinked to deleterious load [38, 39]. Additional models include genetic drift [40–42] and intergenomic conflicts arising from meiotic drive or other selfish elements [43, 44], and testing among all of these hypotheses has been difficult because few predictions are unique to only one model and genes potentially causing these shifts are difficult to identify [45]. Nonetheless, in some cases, the models make different predictions for the patterns of turnover. For example, the deleterious mutation load model predicts that switches in heterogamety between XY and ZW systems should be uncommon because this results in fixation of the Y or W chromosome as an autosome and expression of the homozygous deleterious load [39, 46]. Models associated with arms races, such as the intergenomic conflict models, predict that the length of the sex determination pathway should increase with each successive wave of driving elements [19, 47]. Finally, the hot-potato model predicts a limit to the amount of genetic load that builds up on extant non-recombining regions [38, 39].

**Fig. 1:**
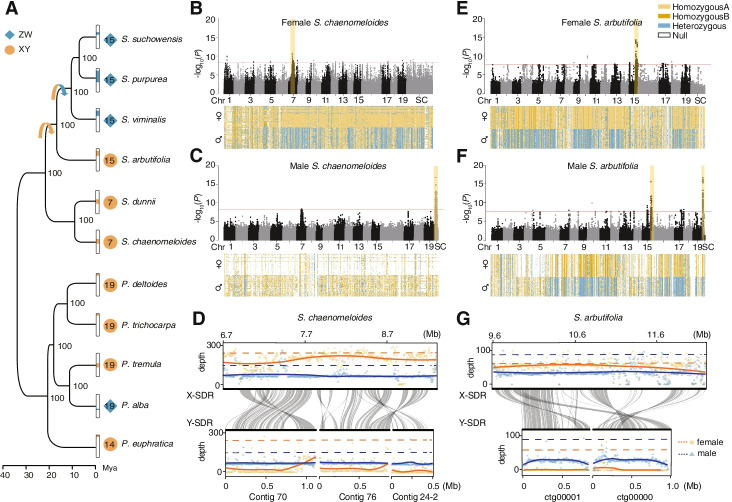
The phylogeny of *S. chaenomeloides* and *S. arbutifolia* and identification of the sex determination regions. **A** Phylogenetic relationship of *S. chaenomeloides* and *S. arbutifolia* and other Salicaceae species, using *Arabidopsis thaliana* as an outgroup. The numbers at the nodes indicate support values based on 100 bootstrap replications. The tree is marked with the type of sex-determining system (orange circles: XY, blue diamonds: ZW), sex chromosomes (numbers within the shapes), and the approximate positions of SDRs on the sex chromosomes. Manhattan plots of *S. chaenomeloides* (**B, C**) and *S. arbutifolia* (**E**, **F**) based on the results of genome-wide association study when the female (**B, E**) and male (**C, F**) genome was used as reference. The *y*-axis represents the strength of association (−log_10_(*P*-value)) for each SNP sorted by chromosomes and scaffolds (SC; *x*-axis). The red line indicates the significance after Bonferroni multiple testing correction (*α* < 0.05). The yellow shadow represents the SDRs, where the genotypes of all SNPs in female and male individuals are colored according to their homozygosity or heterozygosity states. Collinearity relationships between X- and Y-SDR of *S. chaenomeloides* (**D**) and *S. arbutifolia* (**G**). The reads mapping depth of the female and male corresponding to these areas were displayed above and below the collinearity. The orange and blue lines represent female and male respectively. Solid lines represent the mapping depth across these regions and dotted lines represent the genome-wide average depth

*Populus* (poplars) and *Salix* (willows; Salicaceae) are sister genera that are almost entirely dioecious [48–51], and their high diversity of homomorphic XY and ZW sex chromosomes [4, 25, 27, 29, 52–57] is ideal for studying the evolutionary forces driving sex chromosome turnover in plants. Recent studies have shown a wide range of diversity and structural complexity in the sex determination systems of Salicaceae species, especially in poplars where both XY systems on chromosome (chr) 14 and 19, and a ZW system on chr19 have been reported [4, 25, 27, 30, 53, 56]. In contrast, sex determination regions (SDRs) were previously identified on chr15 with a ZW system in multiple willow species of subgenus *Vetrix* (including *S. purpurea*, *S. viminalis* and *S. suchowensis*) [54, 55, 57, 58] and *S. triandra* of subgenus *Salix* [59], until the recent identification of the XY system on chr7 of two willow species from subgenus *Protitea* (*S. nigra* and *S. dunnii*) (Fig. 1A) [29, 52].

Based on the comparison of the haplotype-resolved assemblies of SDRs among poplar species, a general model has been proposed to explain how the XY and ZW systems control sex determination through expression regulation of a type A cytokinin response regulator gene, *RR*, whose function was recently confirmed by CRISPR-Cas9-induced mutation [25, 27]. The *RR* gene triggers female development when expressed and male development when silenced or absent. In poplar species with a ZW sex determination system, the intact *RR* gene was detected in the W-SDR but not in the Z-SDR. Whereas in the XY sex determination system, the intact *RR* gene is located on the autosomal or pseudo-autosomal region of chr19 but not in the SDR, and *RR* partial duplicates in the Y-SDR may act as female suppressors by encoding a siRNA that targets the intact *RR* gene, possibly through RNA-directed DNA methylation [25, 27]. In two willow species (*S. purpurea* and *S. viminalis*), intact *RR* genes have also been detected in the W-SDRs of chr15 [54, 57], which suggests that *Salix* may have the same sex determination mechanism as *Populus*. Because the non-recombining SDRs are large (approximately 2–7.4 Mb with 111-488 genes) in willows, they offer the opportunity to test among evolutionary forces that drive their sex chromosome turnover [29, 52, 54, 57], in contrast to the short SDRs (approximately 33-658 kb) with fewer protein-coding genes (4-37) usually found in poplars [4, 25, 27, 53, 56]. Although the poplar SDRs are generally smaller, there is no indication that specific SDRs are younger than in willows, as they are shared across a wide taxonomic breadth including both Asian and North American species [53, 56, 60].

In this study, we identified and assembled the SDRs of two additional *Salix* species. *Salix chaenomeloides* has an XY SDR on chr7, which is in a similar location as a previously described SDR in *S. nigra* [29] and *S. dunnii* [52]. All three of these species are in subgenus *Protitea* [57]. In *S. arbutifolia* of the subgenus *Chosenia* [61], we found a new XY SDR on chr15. These, combined with the previously described ZW-SDR on chr15 in *S. purpurea*, *S. viminalis*, *S. triandra*, and *S. suchowensis* [54, 55, 57, 58], indicate that sex chromosomes have turned over at least twice in the genus (Fig. 1A). Using these species, we test specific hypotheses about whether (1) the sex determination mechanism in *Salix* shares similarities with the genetic mechanism that has been identified in *Populus*, (2) SDRs in *Salix* exhibit evolutionary strata consistent with episodic expansions of the SDRs, (3) the evolutionary ages of the SDRs are younger than the time of their species divergence, and (4) theoretical models, such as “deleterious mutation load” and “sexually antagonistic selection,” can explain the forces driving sex chromosome turnover.

## Results

### Genome assembly and annotation

By combining third-generation long-read sequencing, short-read sequencing and chromosome conformation capture (Hi-C) technologies, we sequenced and assembled the female and male genomes of *S. chaenomeloides* and *S. arbutifolia* (Additional file 2: Table S1). In brief, the genome of a female *S. chaenomeloides* was sequenced and assembled at 247× coverage (98.7 Gb) with Illumina short reads from libraries with insert sizes ranging from 350 bp to 20 kb, and further improved by combining 36× (14.9 Gb) PacBio sequencing data. For the genome of a female *S. arbutifolia*, 54.8 (169×) Gb Nanopore long reads were first assembled into contigs and then polished using Illumina short reads. Finally 90.59 and 93.22% of the sequences of the two genomes were anchored onto 19 chromosomes with Hi-C data, respectively (Additional file 2: Table S1; Additional file 1: Figs. S1-2). For the male genome assemblies of *S. chaenomeloides* and *S. arbutifolia*, 57.9 (173×) and 27.9 (94×) Gb Nanopore long reads were generated and assembled into contigs with N50 sizes of 6.78 and 2.65 Mb respectively, of which 98.61 and 98.65% were anchored onto 19 chromosomes based on the syntenic relationships of their respective female reference genomes (Additional file 2: Table S1). BUSCO [62] analysis revealed that from 92.7 to 97.8% of the 1375 conserved genes were completely covered in our four assemblies (Additional file 2: Table S2). Consensus quality values (QV) obtained from Merqury [63] revealed a QV from 25.96 to 35.23, corresponding to 99.75 to 99.96% of assembly consensus accuracy (Additional file 2: Table S2). And the comparative genomic analysis showed that these assemblies exhibited extensive collinearity with *P. trichocarpa* (Additional file 1: Fig. S3). These results indicate that our assemblies have a high degree of continuity, coverage, and accuracy.

Based on the transcript dataset, homology searches, and de novo prediction, we identified a total of 27,595 protein-coding genes in the male genome of *S. chaenomeloides*, and 29,609 in the female genome of *S. arbutifolia* (Additional file 2: Table S3), containing 95.5 and 94.3% of the complete conserved BUSCO genes respectively (Additional file 2: Table S2). We then identified 1345 one-to-one orthologs across *S. chaenomeloides*, *S. arbutifolia*, 9 other published Salicaceae species (including 4 willows and 5 poplars), and *Arabidopsis thaliana*. Phylogenetic analysis with these orthologous genes confirmed the monophyletic relationship between the sister genera *Salix* and *Populus* (Fig. 1A). In line with previous studies [50, 61, 64, 65], *S. chaenomeloides* and *S. dunnii* of subgenus *Protitea* were identified as sister to the remaining willow clades, while *S. arbutifolia* of subgenus *Chosenia* was sister to *S. viminalis*, *S. purpurea*, and *S. suchowensis* in the subgenus *Vetrix* (Fig. 1A). Since *S. nigra* only has partial plastid sequence data, we constructed phylogenetic analyses of *Salix* based on the 3153 bp plastid sequences and the results showed that *S. nigra* was sister to *S. chaenomeloides* and *S. dunnii* (Additional file 1: Fig. S4). Molecular dating suggested that the basal *S. chaenomeloides* and *S. dunnii* was estimated to diverge from other willow species around 22 million years ago (Mya), while the next successive clade *S. arbutifolia* was estimated to have originated around 17 Mya (Fig. 1A). This high-resolved phylogeny provides a framework for our subsequent study on the dynamic evolutionary history of their sex determination.

### XY sex determination on chromosome 7 in S. chaenomeloides

For *S. chaenomeloides*, we resequenced 30 male and 30 female individuals with an average coverage of 32× (Additional file 2: Table S4) and identified 113 SNPs that were significantly associated with sex (*α* < 0.05 after Bonferroni correction) when using the female assembly as a reference (Fig. 1B; Additional file 2: Table S5). Most sex-associated SNPs (92 SNPs, 81.42%) occurred between 6.39 and 8.73 Mb on chr7. Across these SNPs, about 74.2% of the genotypes were heterozygous in males, while only 7.1% were heterozygous in females (Additional file 2: Table S5). Further analysis showed that the heterozygous genotypes in females were almost exclusively caused by three individuals, for which 69.3% of the genotypes were heterozygous, suggesting occasional recombination events between X- and Y-SDR, the phenomena of which have also been found in other Salicaceae species [4, 29]. When these individuals were excluded, only 0.2% of the sex-associated SNPs were heterozygous in females (Additional file 2: Table S6). Therefore, these results are consistent with an XY system in which males are heterogametic sex.

We also found that the remaining sex-associated SNPs were scattered across various chromosomal locations (Fig. 1B; Additional file 2: Table S5). This pattern of dispersion has also been found in other Salicaceae species and arose because the reference genome is from a homogametic individual (XX or ZZ) and Y- (or W-) limited genes were misaligned. Therefore, we realigned the resequencing data to our assembled male reference genome and recovered 132 sex-associated SNPs, all of which were located on three unanchored contigs (Fig. 1C; Additional file 2: Table S7). Surprisingly, we found that 73.36% of the genotypes were missing in females across the 132 sex-associated SNPs, whereas 66.29% were homozygous in males. The most likely cause for this observation is that the three contigs are unique to the Y chromosome of *S. chaenomeloides* and thus no reads from females (XX) mapped to these regions, which resulted in alignment of only reads from Y-specific regions of male individuals that exhibited hemizygous Y genotypes. Consistent with this explanation, we found that these three contigs have a high degree of similarity to the sex-associated region on chr7 of the female genome (Additional file 1: Fig. S5). In addition, these contigs showed extensive collinearity with 6.70 to 8.93 Mb of chr7 of the male reference genome, a region containing 321 SNPs with sex-associated *P*-values less than 1.0×10^−7^, although they were not significant after Bonferroni correction for multiple tests. About 88.45% of the genotypes across these SNPs were homozygous in females, while 95.72% were heterozygous in males, consistent with an XY system (Additional file 2: Table S8). Further evidence supporting our inference resulted from the fact that all these regions exhibited half the sequencing depth of the other genomic regions in males but not females (Fig. 1D). These results strongly indicate that we have assembled the fragments of X and Y chromosomes separately. According to the segregation pattern of sex-associated SNPs and the distribution of sequencing depth, we referred the corresponding region of chr7 (~6.7–9.3 Mb) as X-SDR and the three discrete contigs spanning ~2.5 Mb as Y-SDR in the male reference. Overall, our results clearly demonstrated that *S. chaenomeloides* has an XY sex determination system with the SDR located on a non-terminal region of chr7.

### XY sex determination on chromosome 15 in S. arbutifolia

For *S. arbutifolia*, we resequenced the genomes of 26 male and 30 female individuals with an average coverage of 38× and performed a similar GWAS strategy to identify sex-associated SNPs (Additional file 2: Table S4). When the male assembly of *S. arbutifolia* was used as a reference, a total of 126 SNPs that were significantly associated with sex (*α* < 0.05 after Bonferroni correction) were identified (Fig. 1F; Additional file 2: Table S9). Among these, 61 (48.41%) occurred on chr15 and 53 (42.06%) occurred on an unanchored contig that was collinear with a region of chr15 in the *S. purpurea* genome. Across these SNPs, approximately 89.84% of the genotypes were homozygous in females, and 70.45% were heterozygous in males (Additional file 2: Table S9), a pattern largely consistent with an XY sex determination system. A total of 20.63% of the genotypes were homozygous in males for the reference allele. Similar to the above results, this observation may have resulted from the presence of X-Y chimeric assembly in the male reference. The XY sex determination system with the SDR located on chr15 was also confirmed when the female assembly was used as a reference (Fig. 1E; Additional file 2: Table S10). Since we had not identified obvious Y-specific sequences in the male genome, we combined the male-specific k-mer and the read-based phasing assembly performed by WhatsHap [66] to reconstruct Y-SDR to two contigs with a total length of ~1.8 Mb. This assembly was supported by the male-specific sequencing depth profile and widespread collinearity with the sex-associated regions in the female assembly of *S. arbutifolia* (Fig. 1G).

### Composition and divergence of the Y-SDRs in the two willow species

**Fig. 2:**
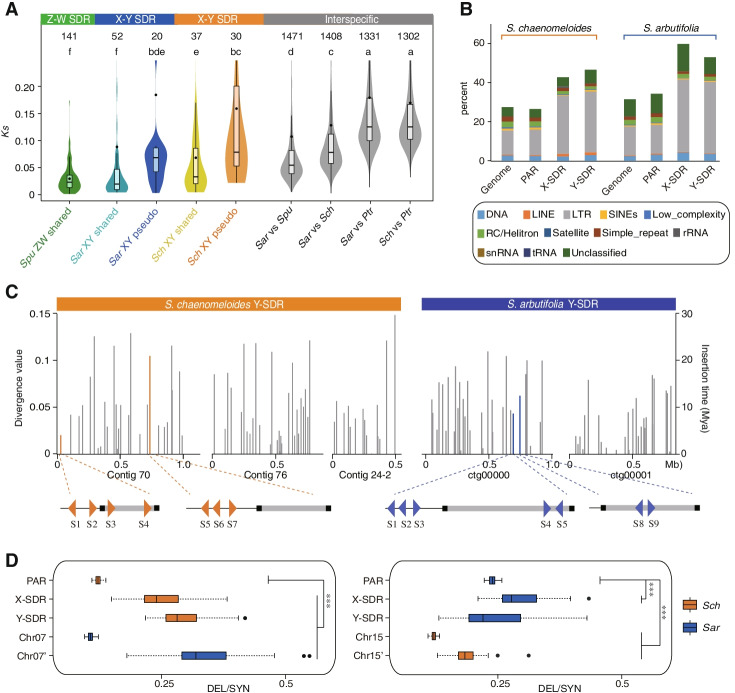
Degeneration of the Y-SDRs. **A** Comparison of the synonymous substitution rates (*Ks*) of genes between X-, Y-SDR, and different species (genes on Chr01). Significant values of the Mann-Whitney *U* test are marked above. *Sar*: *S. arbutifolia*; *Sch*: *S. chaenomeloides*; *Spu*: *S. purpurea*; *Ptr*: *P. trichocarpa*; shared and pseudo: shared and pseudo genes. **B** Types and proportions of the repetitive sequences in each region of *S. chaenomeloides* and *S. arbutifolia*. **C** Estimated divergence values and insertion times of full-length long terminal repeat retrotransposons (LTR-RTs) in Y-SDRs. The orange and blue lines represent LTRs around the *RR* partial duplicates, and their corresponding positions are shown below. **D** Comparison of deleterious mutations in *S. chaenomeloides* (yellow) and *S. arbutifolia* (blue). Chr07 and chr15 represent all genomic regions of the chromosome. Chr07’ represents the autosomal genomic region in *S. arbutifolia* (which has the SDR on Chr15) that is collinear with the *S. chaenomeloides* SDR, and Chr15’ represents the autosomal genomic region in *S. chaenomeloides* (which has the SDR on Chr07) that is collinear with the *S. arbutifolia* SDR. Significant values from the Mann-Whitney *U* test are indicated with asterisks: ***p<0.001. *Sch*: *S. chaenomeloides*; *Sar*: *S. arbutifolia*; DEL: deleterious variants; SYN: synonymous variants

To gain insight into the composition and evolutionary history of the willow SDRs, we predicted 78 and 91 protein-coding genes in the Y-SDR of *S. chaenomeloides* and *S. arbutifolia* respectively (Additional file 2: Tables S11-12). By comparing with their corresponding X-SDRs, we found that the region where these genes are located is also rich in a large number of tandem repeat genes, genes translocated from autosomes, and SDR-specific genes. However, most Y-specific genes had no known function, and no protein-coding genes were homologous between the Y-SDRs of the two willow species. We next estimated the synonymous substitutions (*Ks*) between the shared X-Y homologs to test whether there were evolutionary strata with different degrees of divergence in these two species. A total of 37 pairs of X-Y homologs were identified in *S. chaenomeloides* and the median *Ks* was 0.033 ± 0.014 SE (Fig. 2A). In comparison, the *Ks* between 52 pairs of X-Y homologs in *S. arbutifolia* was 0.020 ± 0.033 SE (Fig. 2A). No obvious evidence was found to support the existence of strata in either of these two species. However, the lower divergence between X-Y homologs of *S. arbutifolia* suggested that its SDR evolved more recently than *S. chaenomeloides*.

In addition, we identified pseudogenes located on the SDRs of these two willows, based on the homology searches and strict filtering criteria for similarity and coverage. We found that the number of pseudogenes on Y-SDRs (22 and 14 respectively) was greater than that of X-SDRs (8 and 8 respectively) for both *S. chaenomeloides* and *S. arbutifolia* (Additional file 2: Tables S11-12). Compared with the X-Y homologs, these pseudogenes exhibited elevated ratios of non-synonymous-to-synonymous substitutions (*Ka*/*Ks*), indicating that they have been undergoing relaxed selective pressure or neutral evolution (Additional file 1: Fig. S6). Interestingly, the *Ks* distribution of the *S. chaenomeloides* pseudogenes was significantly higher than that of X-Y homologs and slightly higher than the divergence between *S. chaenomeloides* and *S. arbutifolia* (Fig. 2A). Similarly, the *Ks* of *S. arbutifolia* pseudogenes was slightly higher than that between *S. arbutifolia* and *S. purpurea* (Fig. 2A). These observations suggested an increase in the rate of nucleotide substitutions in pseudogenes and revealed the gradual and continuous divergence of SDRs after their origination.

### Degeneration of the Y-SDRs in the two willow species

The recombination suppression on the SDR has important evolutionary effects, including accumulation of repetitive elements and deleterious mutations in addition to the loss of gene activity. To study degeneration in more detail, we first annotated repeat sequences in these SDR regions. The results showed that the repeat content of X- and Y-SDR were higher than those of pseudo-autosomal regions (PARs) and other autosomes in both *S. chaenomeloides* and *S. arbutifolia*, especially the content of long terminal repeat (LTR) retrotransposons **(**Fig. 2B; Additional file 2: Table S13). Further analysis identified 71 and 60 intact LTRs in the Y-SDR of *S. chaenomeloides* and *S. arbutifolia*, which were inserted continuously over the last 30 and 23 million years, respectively (Fig. 2C). These results indicated that transposable elements accumulated rapidly in these regions after the establishment of SDRs.

Meanwhile, to explore the accumulation of mutation load in the Y-SDRs, we classified variant bases in coding regions as deleterious (DEL), tolerated (likely to be slightly deleterious, TOL), or synonymous (SYN, assumed to be selectively neutral) (Additional file 1: Fig. S7) based on conservation of the site across diverse plant lineages, using PolyPhen2 [67] and PROVEAN [68]. Using the SYN variants as a selectively neutral reference, the Y-SDR of *S. chaenomeloides* contained significantly higher proportions of DEL and TOL variants than either the X-SDR or the PAR (Fig. 2D; Additional file 1: Fig. S8). However, this pattern was not found in *S. arbutifolia*, whose Y-SDR included more TOL but not more DEL variants than those in X-SDR and PAR genes (Fig. 2D; Additional file 1: Fig. S8). This suggests that the Y-SDR of *S. chaenomeloides* has undergone more genetic degeneration than that of *S. arbutifolia*.

### Exploration of the sex determination mechanism in willows

**Fig. 3:**
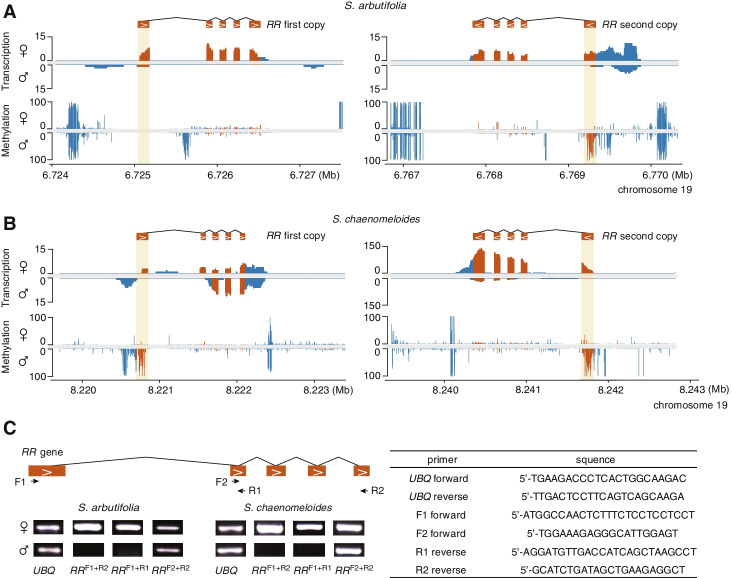
The expression patterns of intact *RR* genes. **A, B** Transcription and methylation levels of the two intact *RR* genes in male and female flower buds of *S. arbutifolia* (**A**) and *S. chaenomeloides* (**B**). The exons and their surrounding areas are shown in orange and blue, respectively. The first exon is marked by yellow shading. **C** RT-PCR amplification of the *RR* transcripts. F1, F2, R1, and R2: forward and reverse PCR primers on exons 1, 2, and 5. The *UBQ* was used as reference gene in both species

Recent studies have shown that the *RR* gene is a master regulator of sex determination in poplars, triggering female development when expressed and male development when inhibited [25, 27]. To study whether the same sex determination mechanism is present in *S. chaenomeloides* and *S. arbutifolia*, we investigated the expression patterns of the intact *RR* gene in their male and female flower buds. We identified two intact *RR* genes on chr19 (autosome) of both species with a nucleotide sequence similarity of more than 98.6%. The RNA-seq data confirmed a female-specific expression of both *RR* genes in flower buds of *S. arbutifolia*, which is consistent with the hypothesis that silencing of the *RR* gene is important for male flower development (Fig. 3A). In contrast, for both copies of the *RR* gene, exons 3–5 were expressed in male flower buds of *S. chaenomeloides*, although the expression level was significantly lower than that of females in the second copy (Fig. 3B). We reverified the alignment of the RNA-seq reads and found that the first exon of the two *RR* genes was specifically silenced in males, a result confirmed by RT-PCR (Fig. 3C). In addition, we found that the first exon and upstream region exhibited a significantly higher methylation level in the male flower buds of *S. chaenomeloides*, especially in the asymmetrical CHH sequence contexts (Fig. 3A, B; Additional file 1: Fig. S9), indicating that the male-specific silencing of the *RR* genes might be due to selective splicing or a shift in transcription start site [69] that is associated with high DNA methylation of the corresponding regions.

**Fig. 4:**
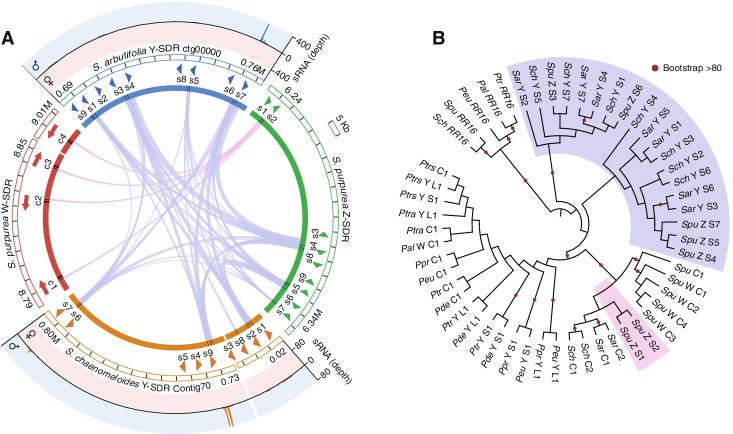
Identification, male-specific expression, and phylogeny of partial *RR* duplicates. **A** The collinearity among the Y-SDRs of *S. chaenomeloides* and *S. arbutifolia*, and Z- and W-SDR of *S. purpurea*. From the inside to the outside: collinearity between the *RR* duplicates, information (position, number, direction) of the *RR* duplicates, and the start and end of the presented part of the SDRs are respectively displayed. In addition, the outermost regions of *S. chaenomeloides* and *S. arbutifolia* also show small RNA alignments along the partial *RR* duplicates and their surrounding 500-bp region in the male and female flower buds. **B** Phylogenetic relationship of the *RR* sequences (including intact genes (“C”) and partial duplicates (“S”: small duplicate; “L”: large duplicate)) identified in the Salicaceae species. The tree was rooted by the paralogous genes “*RR16*.” Abbreviations of all species: *Ptrs*: *P. tremuloides*; *Ptra*: *P. tremula*; *Pal*: *P. alba*; *Ppr*: *P. pruinosa*; *Peu*: *P. euphratica*; *Ptr*: *P. trichocarpa*; *Pde*: *P. deltoides*; *Sch*: *S. chaenomeloides*; *Sar*: *S. arbutifolia*; *Spu*: *S. purpurea*

To verify whether the mechanism found in poplars, whereby *RR* partial duplicates act as female suppressors to suppress the intact *RR* gene, also exists in XY willow species, we searched for the homologous sequences of the *RR* genes on the Y-SDR of *S. chaenomeloides* and *S. arbutifolia*. As expected, partial duplicates of the *RR* gene were identified in both species (Fig. 4A; Additional file 1: Fig. S10). A total of 9 duplicates were found in both species, of which 7 (“SchY:S1-S7”) were homologous to the first exon, and two (“SchY:S8” and “SchY:S9”) to the fourth exon of the intact *RR* genes in *S. chaenomeloides*. In *S. arbutifolia*, a further duplicate (“SarY:S9”) is homologous to the fourth and fifth exons. Consistent with the lack of expression of intact *RR* genes in male flower buds (Fig. 3A–C), a small RNA-seq assay detected small RNAs homologous to these partial duplicates, which may inhibit the expression of the intact autosomal *RR* genes (Fig. 4A; Additional file 1: Fig. S11). Importantly, we found two intact LTRs around the *RR* partial duplicates in both species (Fig. 2C), suggesting that they may be involved in the transposition of the *RR* fragments to SDRs [70, 71]. The insertion times of these two LTRs were estimated to be 21 and 4 Mya in *S. chaenomeloides*, the older insertion of which occurred after its divergence from poplars but close to the diversification of willows. While in *S. arbutifolia*, the insertion times were estimated to be 13 and 9 Mya, both of which occurred after its split with *S. chaenomeloides*, further confirmed that the SDR of *S. arbutifolia* originated more recently than that of *S. chaenomeloides* (Fig. 2C). It should be noted that these estimated times, representing the establishment of the SDRs, may be underestimated, owing to the lack of intact LTR around most of the *RR* partial duplicates and the continuous degradation of the Y-SDRs. Overall, these observations consistently supported similarities between the sex determination mechanism of willow and poplar species, with both involving intact and partial *RR* sequences.

Surprisingly, we also identified 9 *RR* partial duplicates in the Z-linked region of *S. purpurea*, two (“SpuZ:S1” and “SpuZ:S2”) of which were identified previously [25] and showed extensive homology with the four copies of the *RR* intact genes located on the W-SDR of *S. purpurea*, whereas the other 7 duplicates (with “SpuZ:S3-S7” were homologous to the first exon, “SpuZ:S8” to the fourth exon and “SpuZ:S9” to the fourth and fifth exons) showed higher similarity with the *RR* partial duplicates of *S. chaenomeloides* and *S. arbutifolia* than “SpuZ:S1-S2” (Fig. 4A; Additional file 1: Fig. S10). The phylogenetic relationships constructed from all known intact and partial *RR* duplicates of the Salicaceae species indicated that the partial duplicates “SchY:S1-S7,” “SarY:S1-S7,” and “SpuZ:S3-S7,” which are from *S. chaenomeloides*, *S. arbutifolia*, and *S. purpurea* respectively, clustered together and the phylogeny suggests that they originated before the divergence of the intact *RR* genes between willow and poplar (Fig. 4B). In addition, “SpuZ:S1” and “SpuZ:S2” from *S. purpurea* occur together with the intact *RR* genes of the three willow species, suggesting a recent independent origin. Overall, these results indicate that Y-SDR that generates the small RNAs evolved early during the diversification of willows and poplars, and the sex chromosome turnovers in *Salix* species may be the same as in *Populus* by the translocation of the *RR* sequences.

## Discussion

In this study, we determined the sex systems of two willow species and confirmed that, like poplars, willow species also exhibit a fast rate of sex chromosome turnovers. Although previous studies have reported XY system on chr7 [29, 52] and ZW system on chr15 [54, 55, 57] in *Salix*, here we report a new XY system on chr15 and assemble the Y-SDR in the XY species, which allows in-depth investigations of the evolutionary history of *Salix* sex chromosomes. Consistent with previous studies, the Y-SDRs of both willow species were larger than those of poplar. This observation may be explained by the partial overlap of willow SDRs with the centromere [55], selection for recombination suppression between the genes controlling sex determination and genes influencing the more obvious sexual dimorphism in willows [4, 72], or differences in numbers of genes effecting sexual dimorphisms associated with pollination mode between insect-pollinated willows and wind-pollinated poplars. Regardless of the resolution of these speculations, our results reveal enriched diversity of sex determination in willow species and laid the foundation for further research on the dynamic changes of sex chromosomes under a phylogenetic framework.

Our results also suggest that poplars and willows may share similar sex determination mechanisms that involve intact *RR* genes and partial *RR* duplicates. This hypothesis is supported by the presence of *RR* partial duplicates on the Y-SDRs and female-specific expression of *RR* genes in the two willow species (Figs. 3 and 4). Moreover, the phylogenetic positions of these *RR* partial duplicates indicated that they arose before the divergence of *Salix* and *Populus* genera, although the bootstrap support for this pattern was low due to the short sequences (Fig. 4B; Additional file 3: Dataset S1). Nonetheless, the efficacy of this mechanism in willows remains controversial because both the *RR* gene and partial duplicates coexist on the W and Z chromosomes of *S. purpurea* [25, 54]. Future studies in additional willow species or detailed functional studies should provide important insights into the general applicability of this mechanism.

**Fig. 5:**
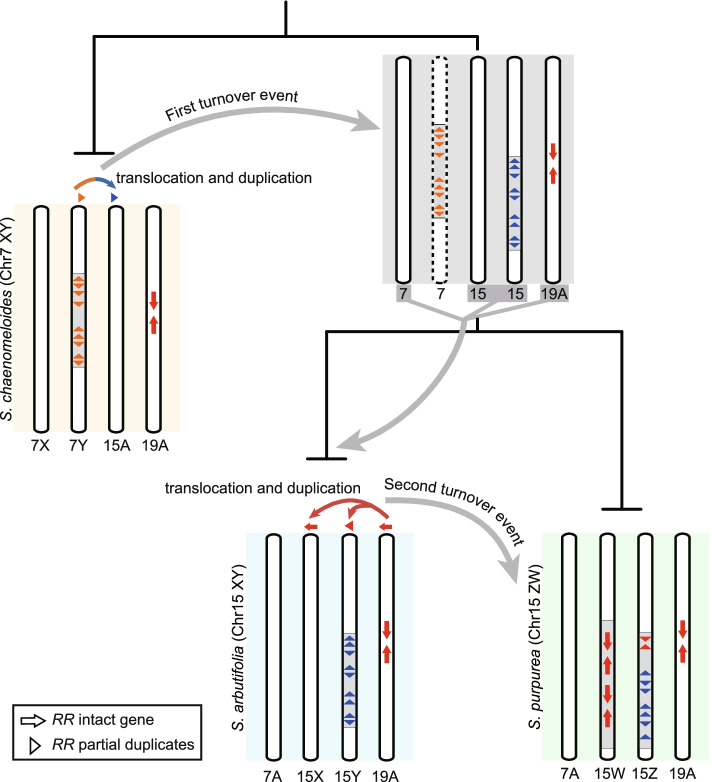
Hypothetical model for two sex system turnover events (7XY→15XY; 15XY→15ZW) in *Salix*. Turnovers may be related to *RR* sequences, including partial duplicates and intact gene

Comparison of the sex systems further revealed that at least two turnover events occurred during the diversification of willow species (Figs. 1A and 5). The species phylogenetic relationships, the divergence of genes and timing of LTR insertion in the SDRs, and the ancient origin of the *RR* partial duplicates (Figs. 1A, 2A, C, and 4B), support the hypothesis of the XY SDR on chr7 as ancestral in the genus *Salix*. The first turnover occurred from chr7 (*S. chaenomeloides*) to chr15 (*S. arbutifolia*), while maintaining an XY system. A second sex chromosome turnover event occurred when heterogamy changed from XY (*S. arbutifolia*) to ZW (*S. purpurea*) on chr15 (Figs. 1A and 5). The transition of sex chromosomes is essentially the movement of new or old sex-determining loci, and our results suggest that sex chromosome turnover in *Salix* may be related to the *RR* sequence. *RR* partial duplicates were translocated from chr7 to chr15 in the first turnover event, and the second turnover event involved the translocation of the autosomal *RR* intact gene from chr19 to chr15 (Fig. 5). Similar translocations of a major sex-determining gene to an autosome resulting in the emergence of a new sex chromosome have also been proposed in some other species [5, 73, 74].

Several theories have predicted evolutionary forces driving sex chromosome turnover, and our results shed light on which may be driving the turnovers in *Salix*. The deleterious mutation model assumes that the accumulation of deleterious mutations in non-recombining regions is expected to lower fitness in heterogametic sexes, thereby promoting sex chromosome turnover to avoid genetic load [38, 39]. Our observations show that the *S. chaenomeloides* Y-SDR carried more pseudogenes and deleterious mutations than *S. arbutifolia* (Additional file 2: Tables S11-12; Fig. 2D), suggesting that genetic degeneration is more advanced in the *S. chaenomeloides* Y-SDR and may have started earlier than in the slightly younger *S. arbutifolia* Y-SDR. This pattern is consistent with the idea that the turnover leading to the chr15 Y-SDR was favored partially because this move purged deleterious loci from the SDR.

Because the sex chromosomes for both *S. arbutifolia* and *S. chaenomeloides* were XY systems, we speculate that this was the ancestral state and that ZW-SDR system is derived from XY. Most previous work has assumed that this is the most common transitional direction for different heterogametic sex systems [37]. For example, experiments in some fish and amphibians confirmed that W is epistatically dominant over Y [75, 76], and sex transitions from XY to ZW has also been observed in multiple species [25, 77, 78]. More interestingly, the highly similar *RR* partial duplicates in the Y-SDR of *S. arbutifolia* and the Z-SDR of *S. purpurea* suggest that the Y became the Z in the second turnover (Figs. 4A and 5). A Z-SDR derived in this manner must be viable when homozygous, which may have been the case soon after the new chr15 Y arose after the first turnover. Alternatively, if the chr15 Y-SDR persisted and accumulated genetic load, this load was overcome during the transition and subsequent evolution of the W. Models have shown that sexually antagonistic selection can drive both switches in heterogamety while the SDR remains in the same location [37] as well as changes in the genomic location of SDRs [36]. For sexually antagonistic selection to drive the two turnover events of *Salix*, the locus under sex-antagonistic selection on chr15 must have been linked to the sex-determining locus (*RR* sequences), resulting in males with higher fitness than those carrying the original Y chromosome. At this time, we have no candidates for sexually selected loci that may have driven this turnover.

Although deleterious mutations and sexually antagonistic selection models may contribute to sex chromosome turnover in *Salix*, other evolutionary forces, such as genetic drift [41, 42, 79] and/or biased sex ratios [44, 80, 81], still cannot be ruled out. Some mechanisms involve special biological conditions, such as small population size, and may require further understanding of population history. At the same time, the combined effect of multiple mechanisms should also be considered. In summary, our results indicated that the rapid rate of sex chromosome turnovers in Salicaceae was driven by a variety of evolutionary forces to keep their sex chromosome perpetually young. Further exploration of the mechanisms that drive sex chromosome turnover requires information from more species.

## Conclusions

We determined an XY sex determination system on chromosomes 7 and 15 in *S. chaenomeloides* and *S. arbutifolia*, respectively, and found that they may share similar sex determination mechanisms with poplars that involve intact *RR* genes and partial *RR* duplicates. Further comparison combined with phylogenetic analysis showed that at least two turnover events occurred during the diversification of willow species, 7XY to 15XY and 15XY to 15ZW, which could be partially explained by the “deleterious mutation load” and “sexually antagonistic selection” theoretical models. These results further refine studies on sex determination and sex chromosome evolution in Salicaceae, suggesting that repeated turnovers may be responsible for the limited degeneration of sex chromosomes in willow species.

## Methods

### Library construction and genome sequencing

All *S. chaenomeloides* and *S. arbutifolia* materials used in this study were collected in Hanzhong (Shanxi) and Baishan (Jilin) of China respectively. Fresh leaves of *S. chaenomeloides* and *S. arbutifolia* were collected, and high-quality genomic DNA was extracted using the Qiagen DNeasy Plant Mini kit (Qiagen, USA) according to the manufacturer’s instructions. Different strategies were used for library construction and genome sequencing of these samples. In brief, genomic DNA of male *S. chaenomeloides* and male and female *S. arbutifolia* were size-selected using the BluePippin system (sage Science), processed following the protocol of Ligation Sequencing Kit (LSK108), and sequenced using the Oxford Nanopore Technology sequencer. Subsequently, low-quality and short-length raw reads were removed to obtain high-quality Nanopore long reads. To correct the errors inherent in Nanopore long reads for genome assembly, we also constructed paired-end libraries and sequenced these libraries on an Illumina HiSeq X platform (Illumina, San Diego, CA).

For the female *S. chaenomeloides*, multiple libraries with different insert sizes, including small-insert paired-end (350 and 450 bp) and long-insert mate-pair (2, 5, 8, 10 and 20 kb) libraries, were constructed according to the Illumina protocols and sequenced on an Illumina HiSeq 2500 platform. The Illumina reads were filtered using the program Trimmomatic (http://www.usadellab.org/cms/?page=trimmomatic) under default parameters. We also constructed a PacBio sequencing library for the female *S. chaenomeloides* using a Pacific Biosciences SMRTbell Template Prep Kit following the recommended protocol. The library was sequenced on the PacBio Sequel platform (Pacific Biosciences, USA). In addition, Hi-C (high-throughput chromatin conformation capture) libraries were constructed for the female *S. chaenomeloides* and *S. arbutifolia* following procedures described previously [82], including chromatin extraction and digestion and DNA ligation, purification, fragmentation, and sequenced on an Illumina HiSeq X system.

### Genome assembly

For the male *S. chaenomeloides* and the male and female *S. arbutifolia*, the quality filtered long reads were first corrected using the module “NextCorrect” implemented in Nextdenovo v2.2.0 (https://github.com/Nextomics/NextDenovo), and then assembled into contigs using SMARTdenovo v1.0.0 (https://github.com/ruanjue/smartdenovo) under default parameters. The initial assemblies were further corrected and polished using the program NextPolish v1.0 (https://github.com/Nextomics/NextPolish), by mapping the filtered Nanopore and Illumina reads to the genome using Minimap2 v2.17 [83] and BWA v0.7.17 [84]. For the female *S. chaenomeloides*, a process of hybrid genome assembly was used [85]. Specifically, the Illumina-based de novo genome assembly was first generated by Platanus v1.2.1 [86] with default parameters using the reads from both paired-end and mate-pair libraries. Then the PacBio subreads were used to fill the gap and improve the genome assembly by PBJelly v14.9 [87] with default parameters.

To construct chromosome-level assemblies, the Hi-C data were mapped to the female assemblies using BWA v0.7.17 [84] and the uniquely mapped reads were retained. The initially assembled contigs were then clustered and extended into chromosomes by using LACHESIS software [88]. To assess the quality of assembly, Hi-C data were mapped to chromosomes using HiC-Pro v2.7.1 [89]. The placement and orientation errors exhibiting obvious discrete chromatin interaction patterns were manually adjusted. For male assemblies, the software RAGOO [90] was used to cluster, order, and orient the assembled scaffolds into 19 pseudo-chromosomes based on a Minimap2 [83] alignment of these scaffolds to their female assemblies. Finally, BUSCO v3.0 [62] analysis was performed to assess the completeness of genome assembly using the “embryophyta_odb10” database with default parameters.

### Gene prediction and annotation

We performed gene prediction on the genome assemblies of the male *S. chaenomeloides* and the female *S. arbutifolia*. Before that, the repetitive elements were first identified using RepeatMasker (open-4-0-7) [91] for detection of known repeats, and RepeatModeler (open-1.0.11) [92] for de novo prediction. Using the repeat masked assemblies, three different approaches were used for gene prediction. Augustus v3.2.3 [93] was used for de novo prediction of protein-coding genes. For homolog-based prediction, the protein sequences from *S. suchowensis* [94], *S. purpurea* [55], *P. trichocarpa* [95], *P. pruinosa* [96], and *P. alba* var. *pyramidalis* [85] were aligned to the genomes using TBLASTN v2.6.0 [97], and the gene models were then predicted using GENEWISE v2.4.1 [98] within the aligned genomic regions. For transcriptome-based prediction, we assembled the RNA-seq using Trinity v2.6.6 [99], and aligned the assembled transcripts to the genomes and filtered with PASA v2.3.3 (Program to Assemble Spliced Alignment) [100] to detect likely coding regions. Finally, the program EvidenceModeler v1.1.1 [101] was used to integrate all predicted gene structures into a consensus set. The predicted genes were functionally annotated by aligning them to Swissprot, TrEMBL [102], and InterPro [103] protein databases with BLASTP v2.6.0 [104]. Gene ontology were assigned by the Blast2GO pipeline [105].

### Phylogenetic analysis

We conducted a phylogenomic analysis for the published genomes of Salicaceae species, including 6 willows (*S. chaenomeloides*, *S. arbutifolia*, *S. suchowensis* [94], *S. purpurea* [54] and *S. viminalis* [57], *S. dunnii* [52], 5 poplars (*P. deltoides* [53], *P. trichocarpa* [95], *P. tremula* [106], *P. alba* [85], *P. euphratica* [107]), and using *Arabidopsis thaliana* [108] as an outgroup. The homology of protein sequences and gene families among all species were determined using OrthoFinder v2.3.11 [109]. A maximum likelihood (ML) phylogenetic tree was then constructed by RAxML v8.2.11 [110] using the single-copy orthologs, and the divergence times among species were estimated using the MCMCtree program [111]. Since *S. nigra* only has partial plastid sequence data, we collected the published partial sequences of matK (KM002266), rbcL (AB012790), rpoB (KM002594), and rpoC1 (HQ594123) and performed phylogenetic analysis together with the corresponding homologous sequences from other *Salix* species, as described above.

### Population resequencing and the identification of sex-associated SNPs

Fresh leaves collected from female and male individuals were used for genome resequencing. Genomic DNA of each sample was extracted using the Qiagen DNeasy Plant Mini kit (Qiagen, USA). Sequence libraries were constructed according to the Illumina library preparation pipeline and sequenced on the Illumina HiSeq X platform. After quality control of the raw reads and removal of low-quality reads, the remaining clean reads were aligned to the genome assemblies using BWA v0.7.17 [84]. Polymorphic variants were jointly called for all individuals using Genome Analysis Toolkit (GATK) v3.8.0 [112]. The low-quality variants were removed using the following criteria: (i) variants with a quality score < 30; (ii) SNPs with more than two alleles; (iii) SNPs within 5 bp from any indels; (iv) SNPs with extremely low (< 1/3 average depth) or high (>3 average depth) coverage. The genome-wide association study (GWAS) was performed using PLINK v1.07 [113], and the variants with association at *α* < 0.05 after Bonferroni correction for multiple testing were identified as the significantly sex-associated SNPs.

### Identification and construction of Y-contigs

For *S. chaenomeloides*, we identified three contigs that clearly contain Y-linked sequences in its male genome, based on the following evidence: (i) most of the sex-associated SNPs in these contigs were homozygous in males, but missing in females; (ii) they were widely collinear with the sex-associated regions on chromosome 7 in both the male and female genomes; and (iii) the depth of coverage on these contigs was male specific and was half that of other genomic regions. For *S. arbutifolia*, due to the lack of clear Y-contigs in the male genome, we developed a pipeline to de novo assemble its Y-contig. In brief, we first mapped the corrected Nanopore reads from the male individual to the reference genome using minimap2 program [83] and generated the linked SNPs that were present in two different haplotypes by the software WhatsHap [66]. The linked SNPs on chromosome 15 were further used for identification of X or Y-specific SNP blocks. Nanopore reads that contained variants located on the Y phased SNP blocks were then clustered into reads from Y chromosome. In addition, we also selected all the 30-bp k-mers from male and female individuals using Jellyfish v2.2.9 [114], identified the male-specific k-mers that were absent in females and present in at least 20 male individuals, and searched for the Y Nanopore reads that contain at least one of the male-specific k-mers. Finally, these Y reads obtained from WhatsHap and male-specific k-mers were deduplicated and assembled into two contigs using the software Nextdenovo v2.0 (https://github.com/Nextomics/NextDenovo). The assembly accuracy of the Y-contigs was further confirmed by collinear analysis with X-SDR, and the relative depth of coverage between male and female individuals using the above criteria. The repeat annotation and gene prediction of the Y-contigs were performed using the methods described above, and the partial *RR* duplicates in the Y-contigs were identified by BLAST [104] using the intact *RR* sequences as queries. The program MUSCLE implemented in MEGA7 [115] was used to align the first exon sequences of the intact and partial *RR* duplicates of all available Salicaceae species, and the maximum likelihood tree was constructed using MEGA7 [115] with default parameters.

### Comparison of X- and Y-SDR

To identify homologous genes on the X- and Y-SDRs, we performed a reciprocal BLAST [104] of all annotated sequences in these regions with default parameters. Tandem duplications were identified as genes with expectation value of 1 × 10^−10^ that occurred within a 500-kb window. All these genes were classified according to the following criteria: (i) if their homologous genes were found on the other haplotype, the genes of one haplotype were considered “X-Y shared”; (ii) those with hits to the corresponding chromosomal region of the other Salicaceae genomes were designated as “Ancestral” under the assumption that the homolog was present prior to the establishment of the SDR; (iii) those genes that lacked hits to the corresponding region in either species, but had a mutual best hit to an autosomal gene, were designated as “autosomal transpositions”; (iv) for genes without homologs between X and Y-SDRs, we further tried to annotate their pseudogenes manually in the corresponding haplotype when the sequence identity >70% with the true genes, and the genes that had a corresponding pseudogene and/or an “Ancestral” homolog were designated as “lost,” whereas the remaining genes were designated as “specific.” Note that these criteria are not mutually exclusive, so some genes may have multiple classifications. The non-synonymous (*Ka*) and synonymous (*Ks*) substitution rate and their ratio were estimated using the yn00 function in PAML [111].

### Expression of intact and partial RR duplicates in flower buds

The male and female flower buds of *S. chaenomeloides* and *S. arbutifolia* were collected in Hanzhong, Shaanxi, and Baishan, Jilin, China, in September and October 2020, respectively. To determine the expression patterns of the intact *RR* duplicates, the total RNA was extracted and purified using Plant Total RNA purification kit with DNase I (Aidlab), and reverse-transcribed using a Tiangen Fast Quant RT Kit (Tiangen). Real-time PCR (RT-PCR) was then performed using the forward (*UBQ*: 5′-TGAAGACCCTCACTGGCAAGAC; *RR*^F1^: 5′-ATGGCCAACTCTTTCTCCTCCTCCT; *RR*^F2^: 5′-TGGAAAGAGGGCATTGGAGT) and the reverse (*UBQ*: 5′-TTGACTCCTTCAGTCAGCAAGA; *RR*^R1^: 5′-AGGATGTTGACCATCAGCTAAGCCT; *RR*^R2^: 5′-GCATCTGATAGCTGAAGAGGCT) primers under the conditions: 5 min at 95°C, 36 cycles of 30s at 95°C, 20s at 55°C, 25s at 72°C and a final extension step of 3 min at 72°C.

We also constructed cDNA libraries for each sample using the RNA Library Prep Kit for Illumina according to the manufacturer’s instructions (NEB, USA). The libraries were sequenced on an Illumina HiSeq X platform and the obtained clean reads were mapped to the reference genome using HISAT2 v2.1.0 [116]. Only reads with a “uniq” match were used for further analysis. In addition, we used the CTAB method [117] to extract genomic DNA from each sample, and constructed whole genome bisulfite sequencing libraries following procedures described previously [118]. All libraries were sequenced on an Illumina HiSeq 2500 platform to an average depth of 50× and the obtained reads were aligned to the corresponding reference genome using the software Bismark v0.22.3 [119]. Finally, the different contexts of methylation (GpG, CHG, and CHH) were extracted and merged. And the conversion rates were > 99.26% for all libraries estimated by aligning reads to the unmethylated chloroplast genome. In order to detect the expression of *RR* partial duplications, small RNA libraries were constructed and sequenced using the DNBSEQ platform (BGI, Shenzhen, China). The obtained reads were mapped to the male genome of *S. chaenomeloides* and *S. arbutifolia* using Bowtie v.1.2.2 [120] with no mismatch allowed and only reads with a “uniq” match were used to determine the expression of partial *RR* duplicates.

### Annotation of full-length LTR-RTs and estimation of insertion times

To detect recent insertions of transposable elements within the Y-SDRs, LTRharvest [121] and LTRdigest [122] were used to de novo predict the full-length LTR-RTs. We estimated time since transposition based on the number of substitutions between the two LTR arms [123, 124]. To estimate the substitution rate between the flanking LTR repeats, 5′ and 3′ repeats of each LTR retrotransposon were aligned by MUSCLE using the default parameters provided in MEGA7 [115]. The divergence values were then corrected for saturation by Kimura’s two-parameter method [125], and insertion times were finally estimated by assuming a mutation rate of 2.5×10^−9^ per year [126].

### Accumulation of deleterious variations in SDRs

We classified variants in the coding regions with respect to their effect on the amino acid sequence [127]. These variants were first classified into synonymous (SYN), missense, and loss-of-function (LOF) using the software SNPEFF [128], the non-synonymous SNPs were then assessed using PolyPhen2 v2.2.2 [67] and PROVEAN v1.1.5 [68] and were finally classified into deleterious (DEL) and tolerated (TOL) with both programs resulting in the same prediction.

## Supplementary Information

Additional file 1: Fig. S1-S11. Supplementary figure legends and supplementary figures.

Additional file 2:Table S1-S13. Supplementary Tables.

Additional file 3:Dataset S1. Multifasta file of all intact and partial *RR* duplicates of the Salicaceae species used for the phylogenetic analysis shown in Fig. 4B.

Additional file 4. Uncropped images of agarose gel electrophoresis in Fig. 3C.

Additional file 5. Peer review history.

## Data Availability

All data needed to evaluate the conclusions in the paper are present in the main text or the Supplementary Information files. All sequence data, genome assembly, and annotation information of *S. chaenomeloides* and *S. arbutifolia* used in this manuscript have been deposited in the National Genomics Data Center (NGDC; https://bigd.big.ac.cn/bioproject) under BioProject accession number PRJCA005435 [129].
