# Supplementary material for: Repeated turnovers keep sex chromosomes young in willows
Source: Genome Biol. 2022 Sep 23;23:200. doi: 10.1186/s13059-022-02769-w (PMC9502649; doi:10.1186/s13059-022-02769-w)
Supplement: Supplementary file 1 — Additional file 1: Fig. S1-S11. Supplementary figure legends and supplementary figures. [file 13059_2022_2769_MOESM1_ESM.pdf]

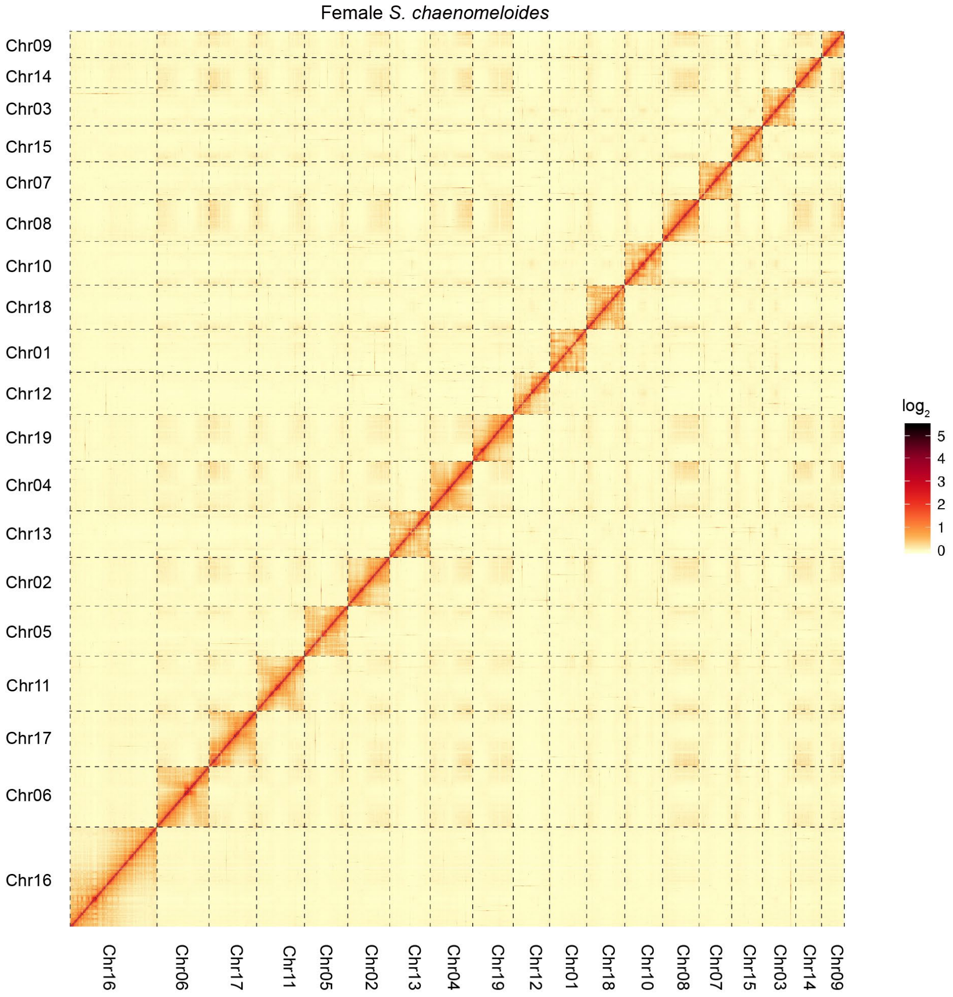

Fig. S1. Heatmap of Hi-C assisted assembly of female *S. chaenomeloides*. Darker red color indicates higher contact probability. The chromosomal order is based on the collinearity with *P. trichocarpa*.

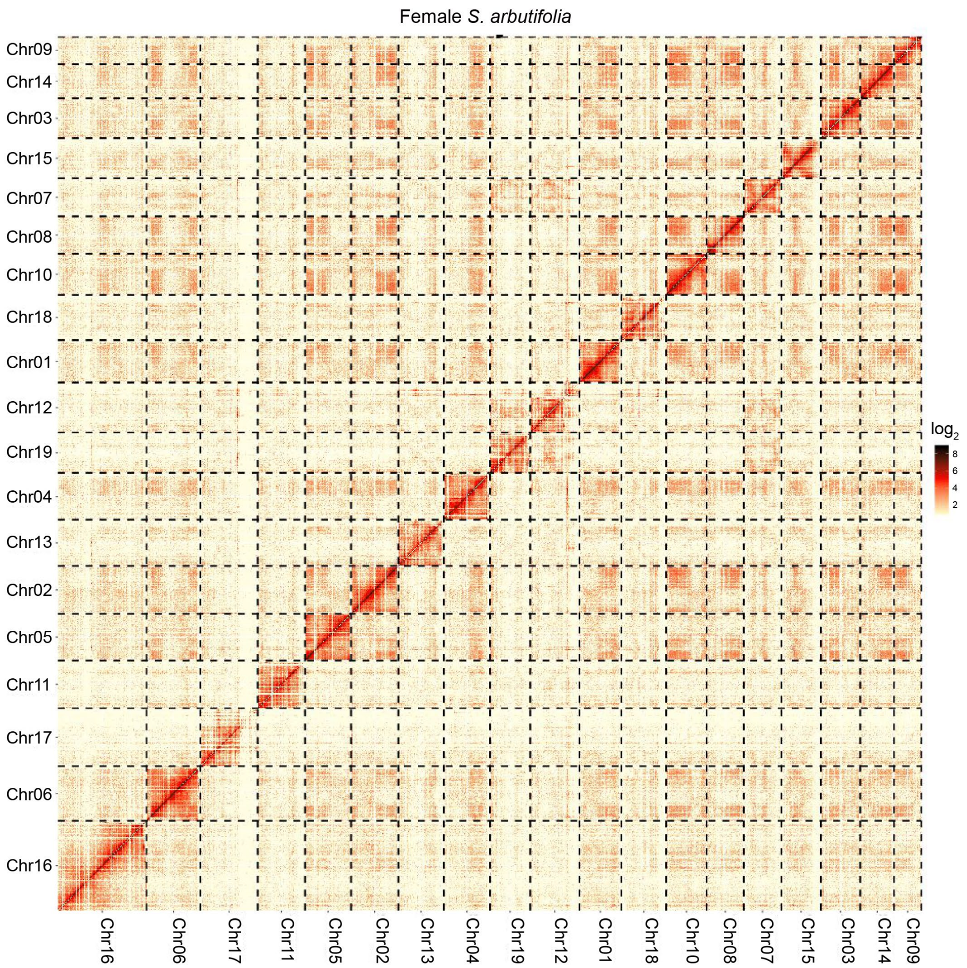

Fig. S2. Heatmap of Hi-C assisted assembly of female *S. arbutifolia*. Darker red color indicates higher contact probability. The chromosomal order is based on the collinearity with *P. trichocarpa*.

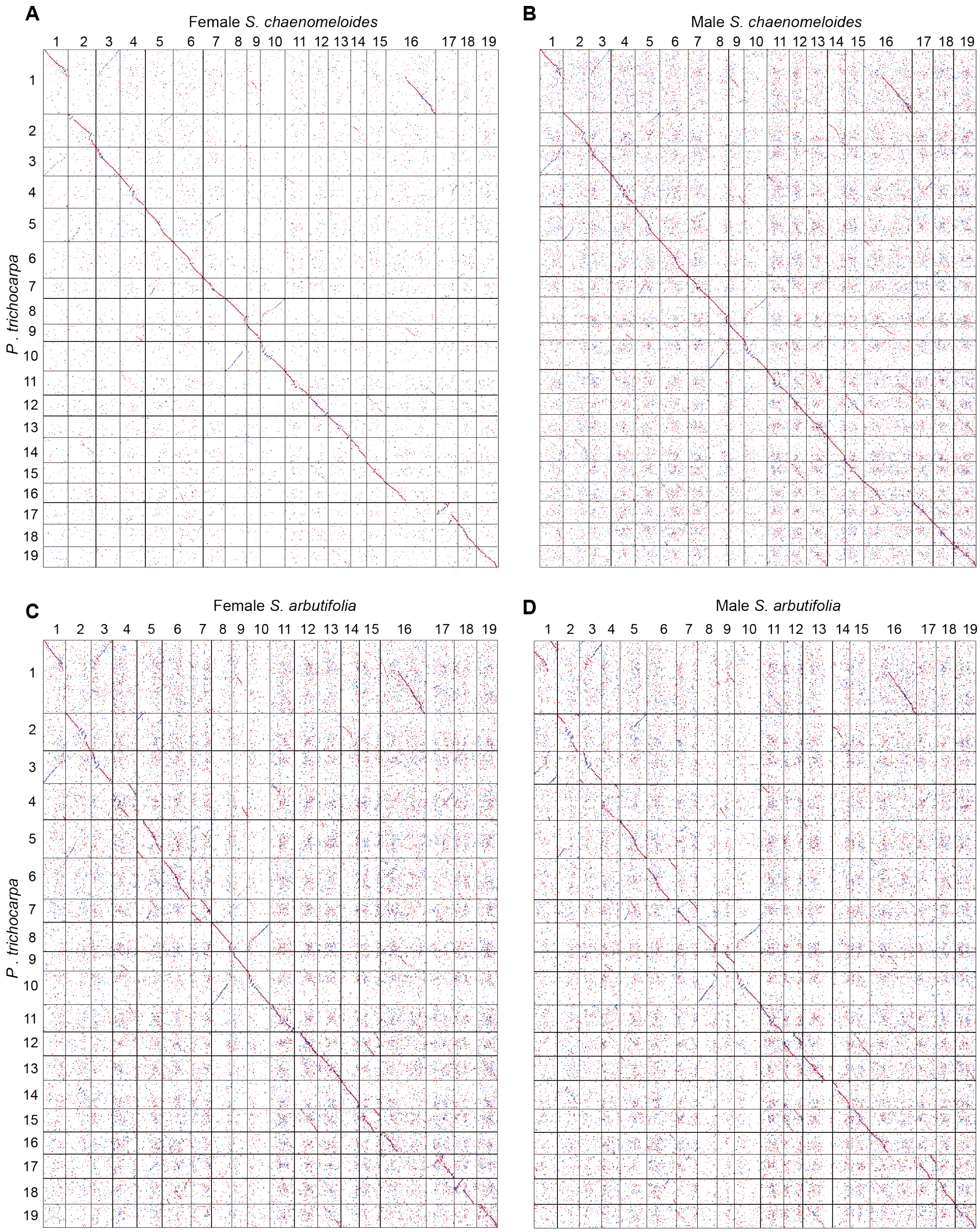

Fig. S3. Genomic synteny between the genomes of female *S. chaenomeloides* (A), male *S. chaenomeloides* (B), female *S. arbutifolia* (C), male *S. arbutifolia* (D) and *P. trichocarpa*.

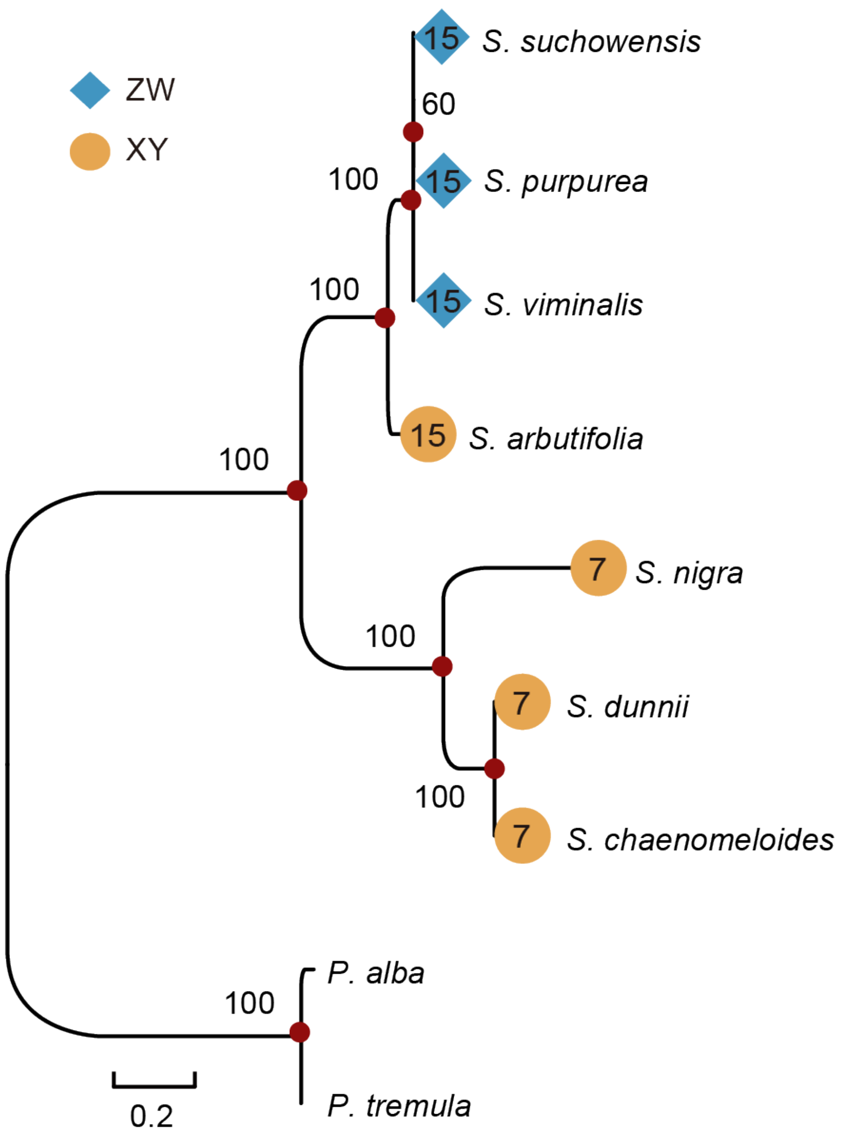

Fig. S4. Phylogenetic relationship of *Salix* species, with *Populus* as outgroup. The numbers at the nodes indicate support values based on 100 bootstrap replications. The tree is marked with the type of sex-determining system (orange circles: XY, blue diamonds: ZW) and sex chromosomes (numbers within the shapes).

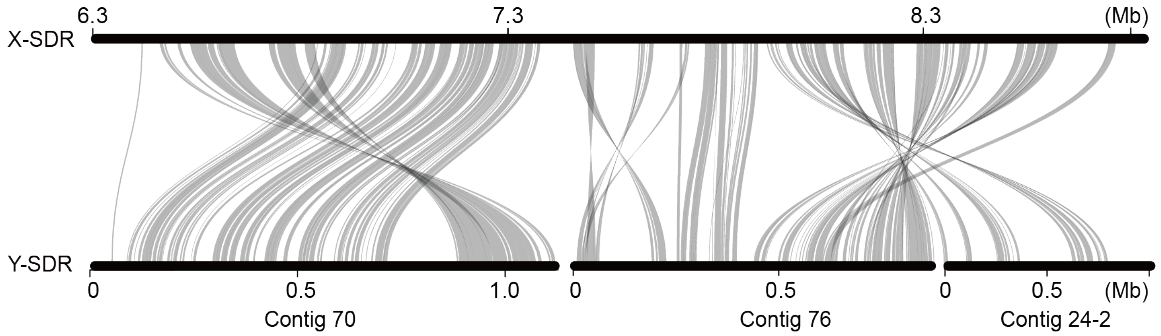

Fig. S5. Synteny relationships of sex-associated regions between reference genomes of male and female *S. chaenomeloides*.

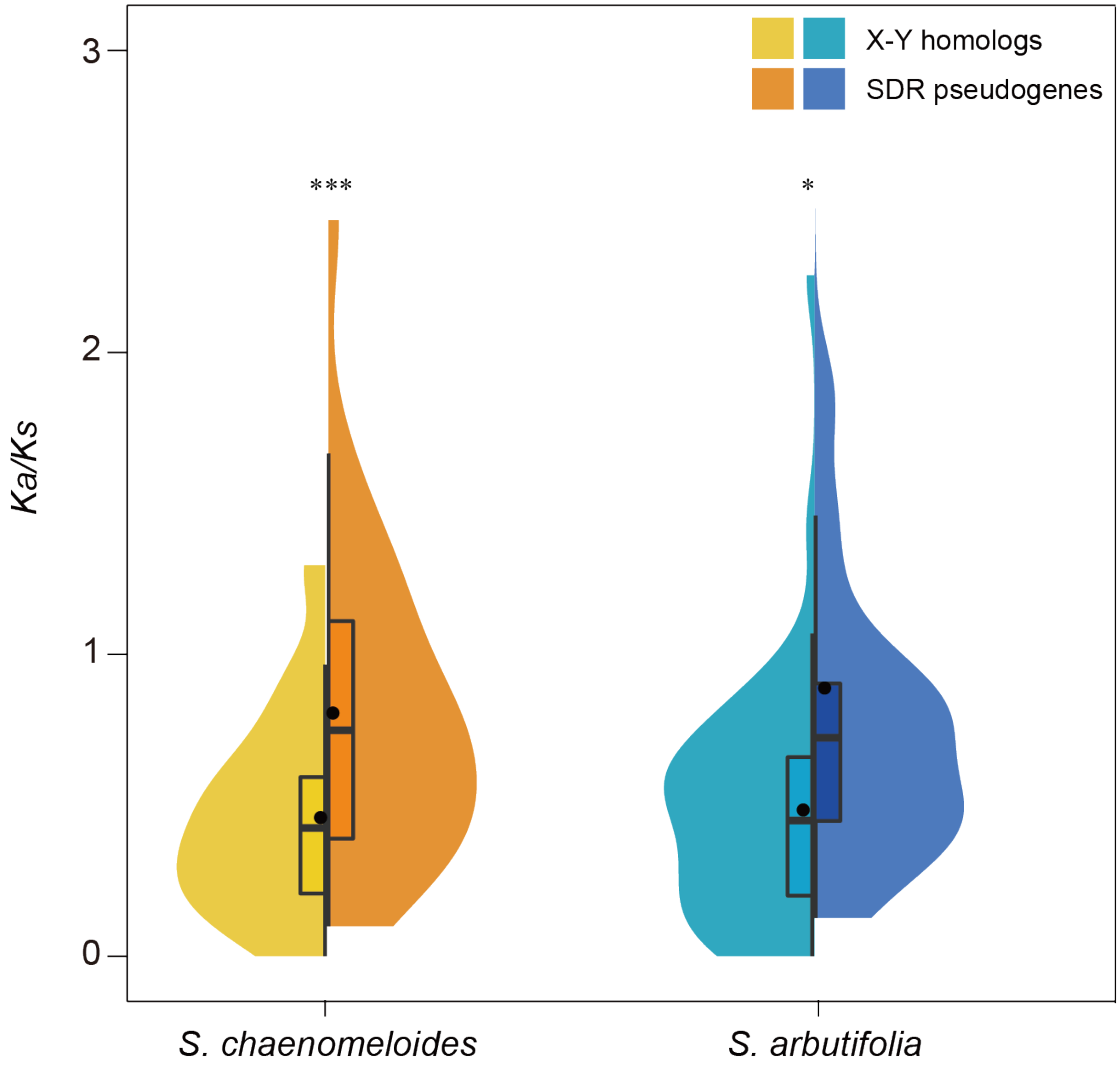

Fig. S6. The ratios of non-synonymous to synonymous substitution rate (*Ka*/*Ks*) for X-Y homologs and pseudogenes in the SDRs of *S. chaenomeloides* and *S. arbutifolia*. Significant values from the Mann-Whitney U test were indicated with asterisks: *$p < 0.05$; **$p < 0.01$; ***$p < 0.001$.

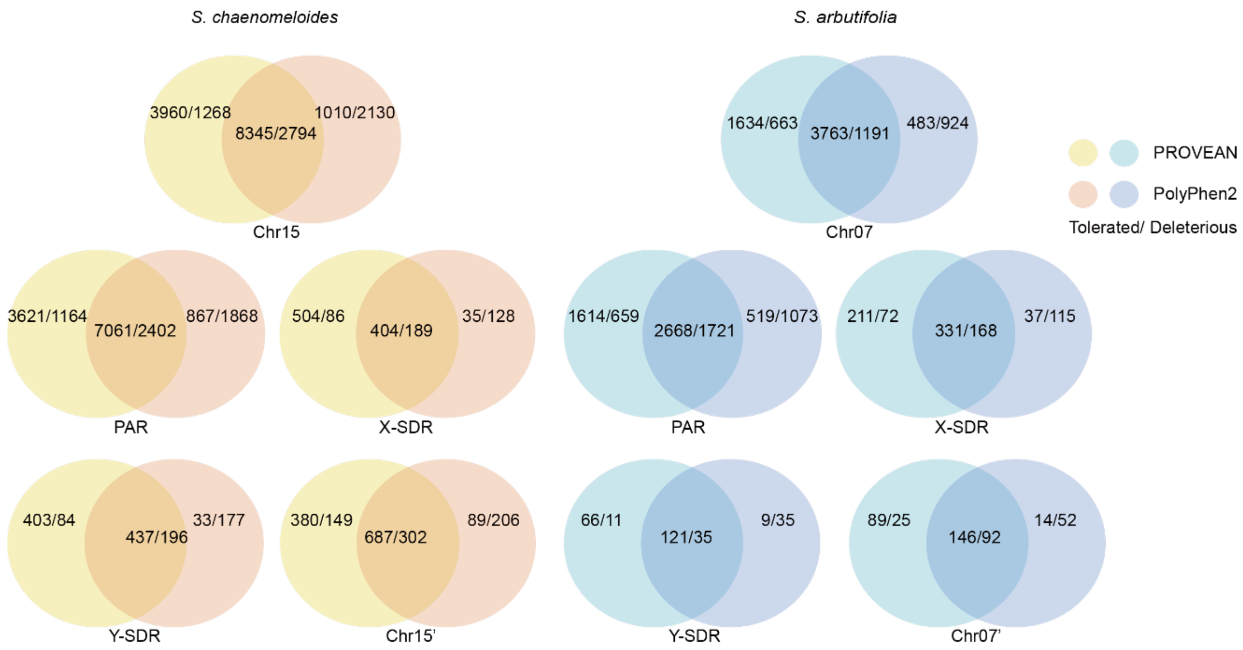

Fig. S7. A Venn diagram of the tolerated and deleterious variants predicted by PolyPhen2 and PROVEAN, for X-, Y-SDRs and pseudoautosomal regions (PARs) of *S. chaenomeloides* and *S. arbutifolia*. Chr7' represents the genomic region in *S. arbutifolia* that is collinear with the *S. chaenomeloides* SDR, and Chr15' represents the genomic region in *S. chaenomeloides* that is collinear with the *S. arbutifolia* SDR.

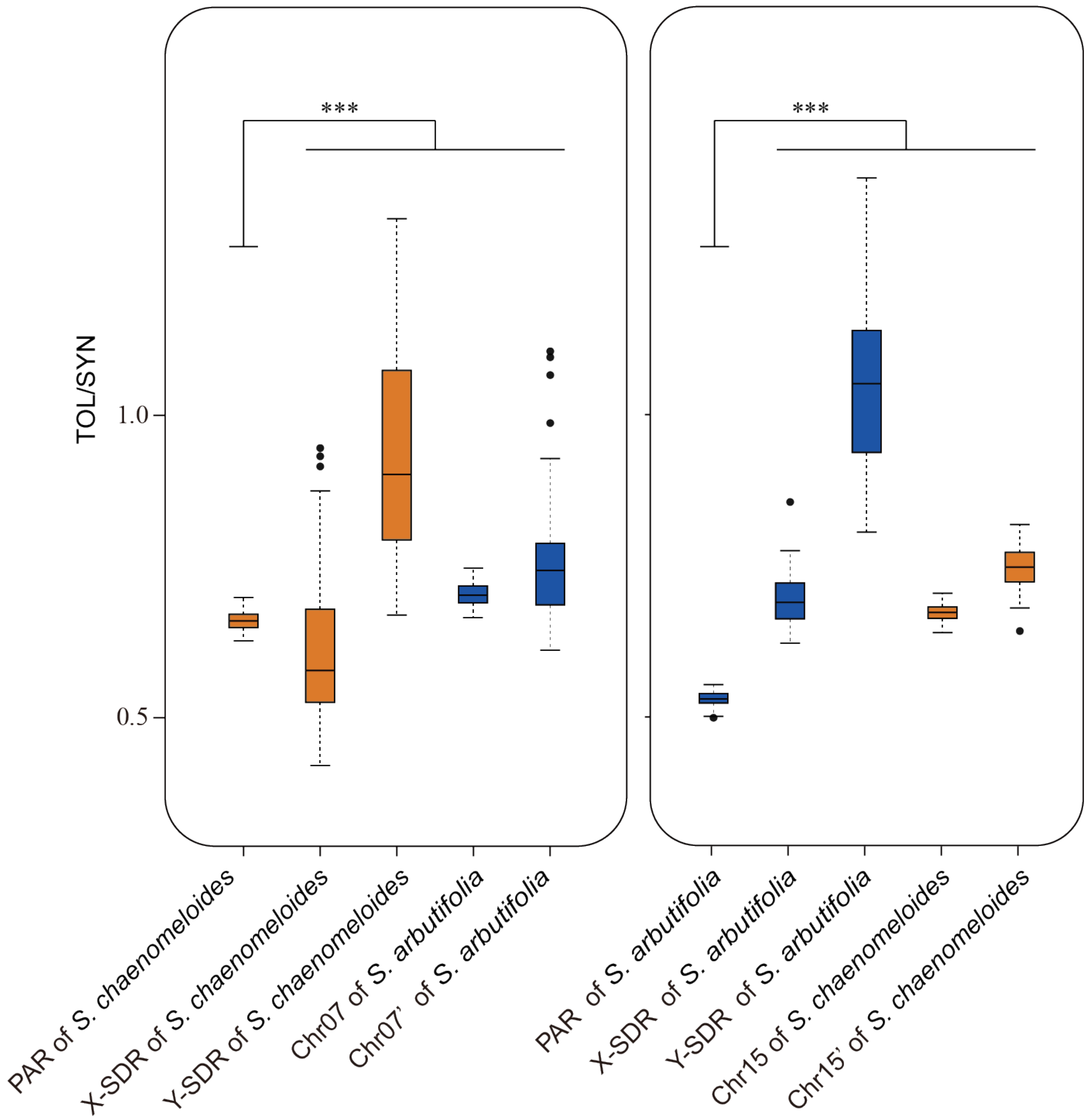

Fig. S8. Comparison of tolerated variation in *S. chaenomeloides* and *S. arbutifolia*. *S. chaenomeloides* and *S. arbutifolia* are represented by orange and blue respectively. Chr07' represents the genomic region in *S. arbutifolia* that is collinear with the *S. chaenomeloides* SDR, and Chr15' represents the genomic region in *S. chaenomeloides* that is collinear with the *S. arbutifolia* SDR. Significant values from the Mann-Whitney U test relative to PAR are indicated with asterisks: ***$p < 0.001$. TOL: tolerated variants; SYN: synonymous variants.

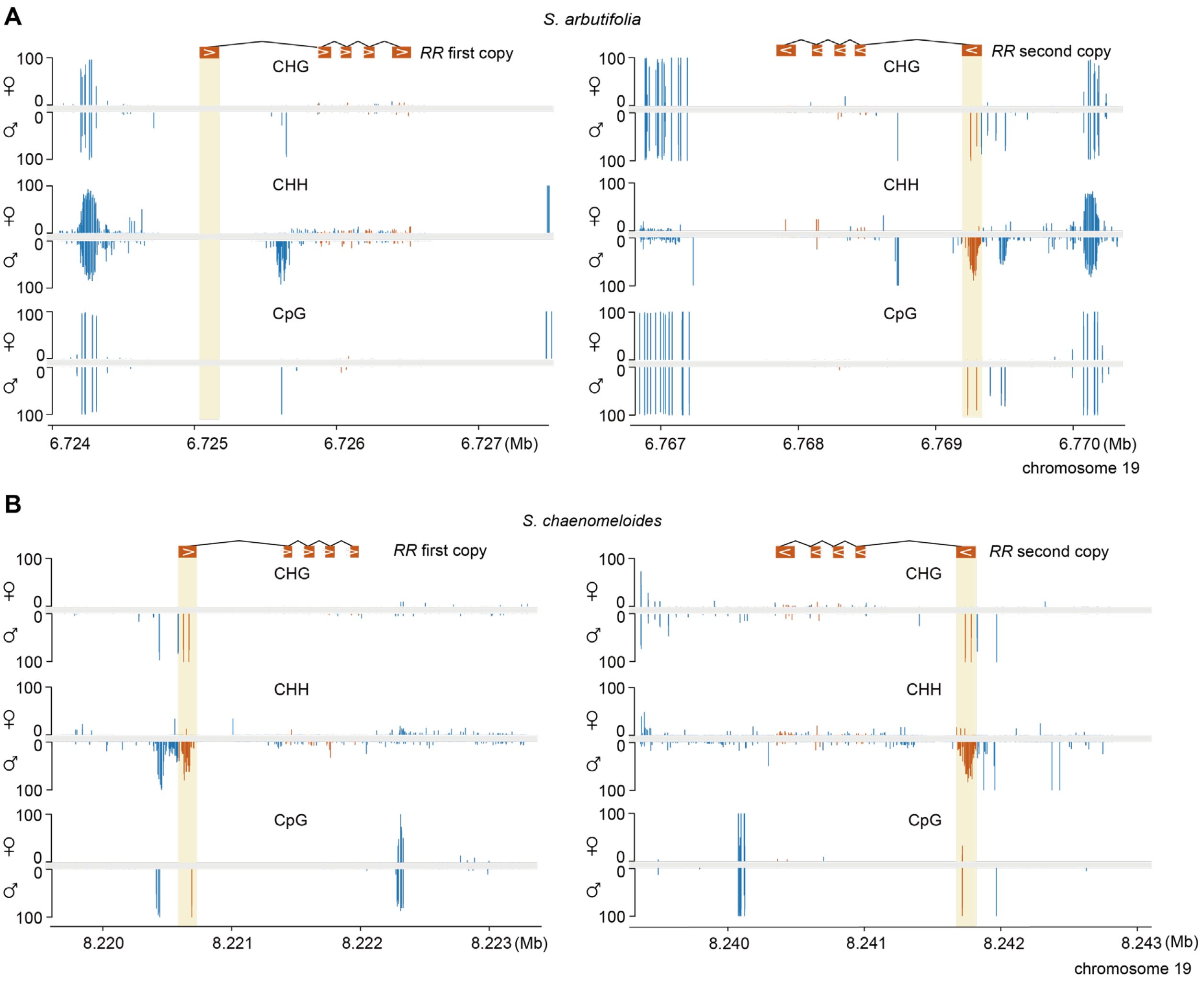

Fig. S9. Different contexts of methylation levels of the two intact *RR* genes in male and female flower buds of *S. arbutifolia* (A) and *S. chaenomeloides* (B). The exons and their surrounding areas are shown in orange and blue, respectively. The first exon is marked by yellow shading.

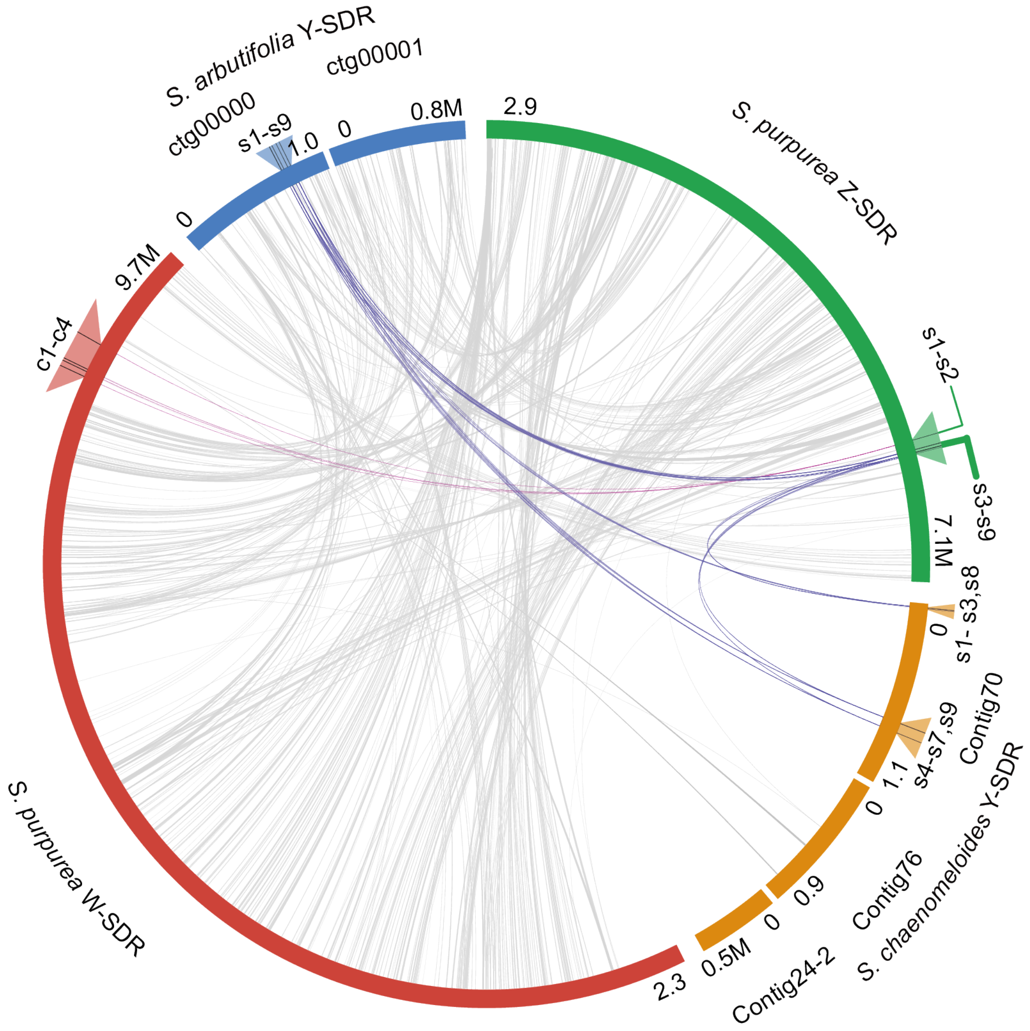

Fig. S10. The gene collinearity among the Y-SDRs of *S. chaenomeloides* and *S. arbutifolia*, and Z- and W-SDRs of *S. purpurea*. The gray line represents the collinearity of protein-coding genes and colored line represents the collinearity of partial *RR* duplicates.

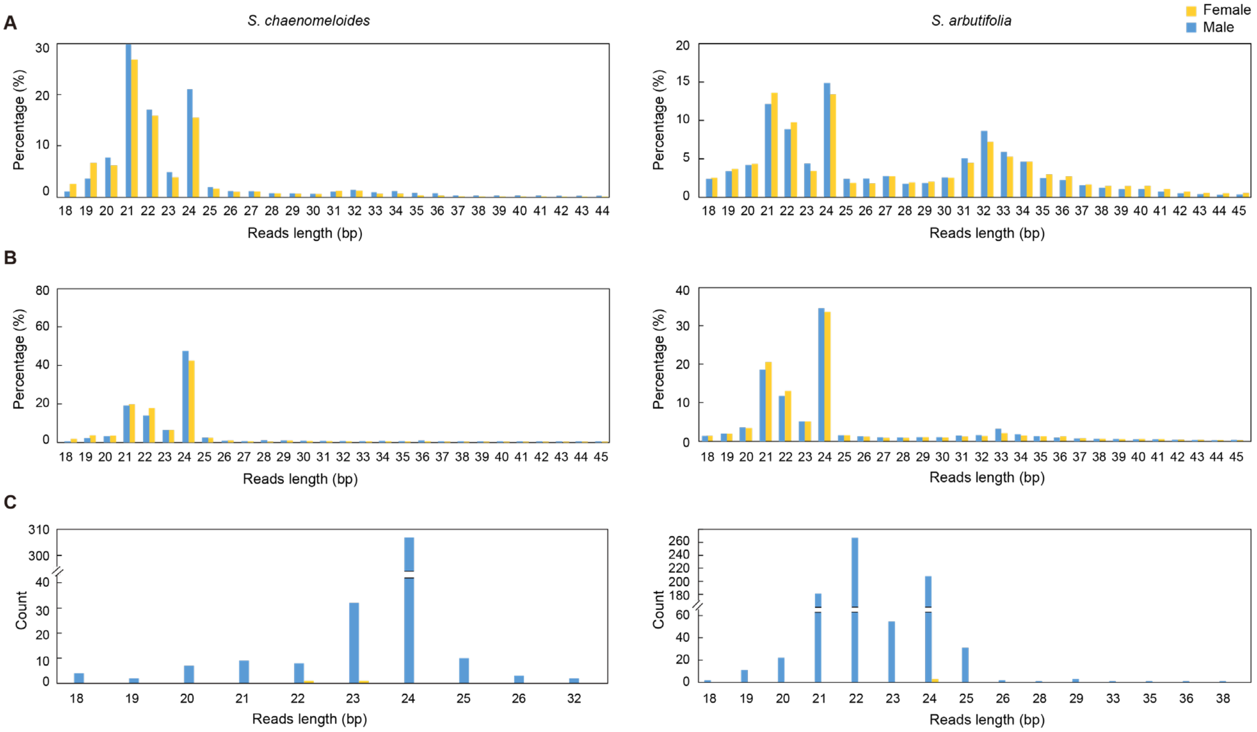

Fig. S11. Length distribution of small RNA reads that are filtered (A), genome-wide aligned (B), and aligned with *RR* partial duplicates and their surrounding 500bp region (C). *S.* *chaenomeloides* and and *S. arbutifolia* are on the left and right respectively. The X axis shows the length of small RNA and the Y axis shows the percentage and number of small RNA with a specific length.
