# Supplementary figures and images for: Repeated turnovers keep sex chromosomes young in willows
Source: Genome Biol. 2022 Sep 23;23:200. doi: 10.1186/s13059-022-02769-w (PMC9502649; doi:10.1186/s13059-022-02769-w)

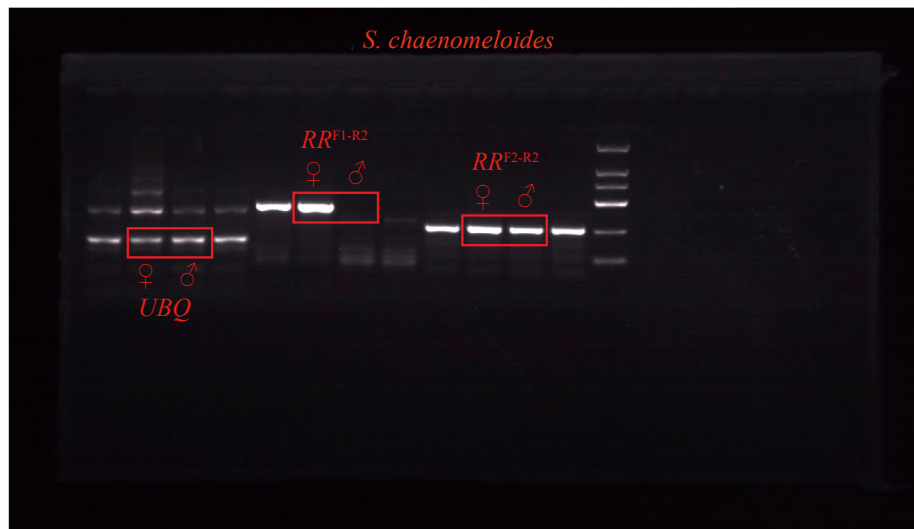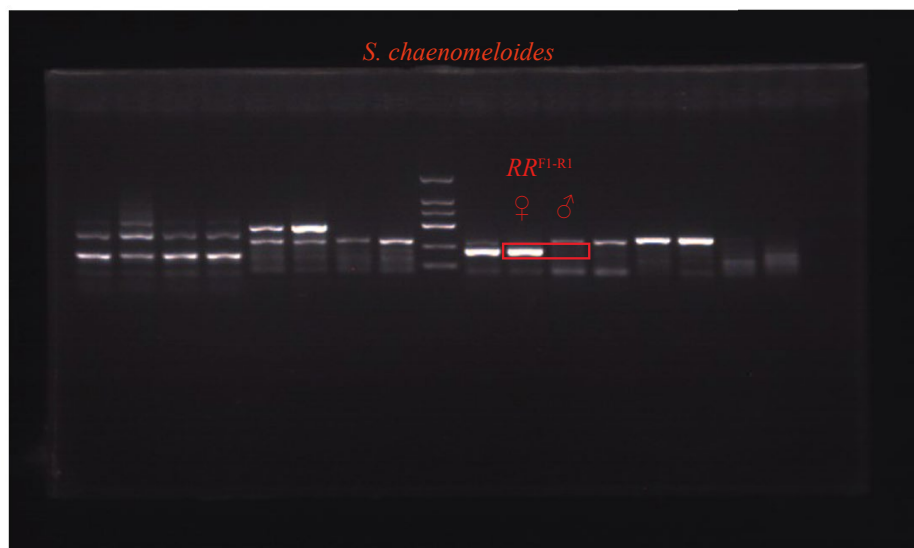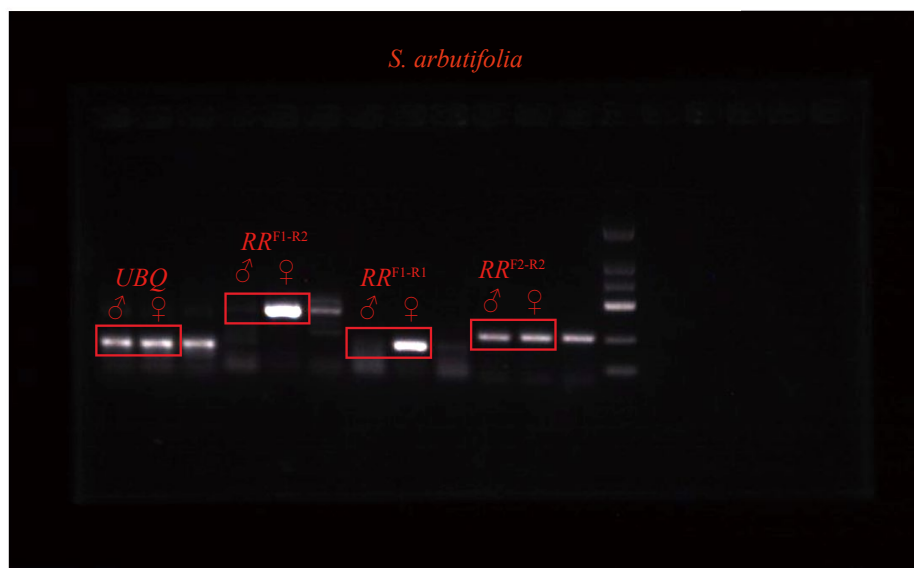

**Additional file 4. Uncropped images of agarose gel electrophoresis in Fig. 3C.**

Supplement: Supplementary file 4 — Additional file 4. Uncropped images of agarose gel electrophoresis in Fig. 3C. [file 13059_2022_2769_MOESM4_ESM.pdf]
