# Supplementary material for: Repeated turnovers keep sex chromosomes young in willows
Source: Genome Biol. 2022 Sep 23;23:200. doi: 10.1186/s13059-022-02769-w (PMC9502649; doi:10.1186/s13059-022-02769-w)
Supplement: Supplementary file 5 — Additional file 5. Peer review history. [file 13059_2022_2769_MOESM5_ESM.docx]

**Review history**

**First round of review**

**Reviewer 1**

Evolution of sex chromosomes in the Salicaceae is particularly fascinating due to frequent sex chromosome turnover events. Wang et al. identified sex determination regions (SDRs) in Salix chaenomeloides and S. arbutifolia through genome-wide association study and performed comparative genomic analysis to trace the evolutionary history of sex chromosomes in Salix species. The manuscript addresses an interesting topic, and the methodological approaches are appropriate. Below are some suggestions for further improvement of the manuscript.

1. Although the authors provided an accession number of the sequence data used in this study, the data is not released yet. All relevant data must be made available before the manuscript is accepted for publication.
2. Pg 3, Line 49-50, please cite research articles, not review articles.
3. Please revise or make the correction for the sentences listed below.
Pg4, Line 69-72; Line 84-87
Pg6, Line 115-117; Line 121-122
Pg9-10, Line 197-201
Pg10, Line 209-211
Pg16, Line 338-341
Pg21, Line 441, please replace "Whatever"
4. Pg5, Line 90-95, please include a reference listed below
Li, W., Wu, H., Li, X. et al. Fine mapping of the sex locus in Salix triandra confirms a consistent sex determination mechanism in genus Salix. Hortic Res 7, 64 (2020).
Li et al. also mapped the sex locus on LG XV in Salix triandra.
5. In the figure legend of Fig. 1G, "red" needs to be corrected.
6. In the figure legend of Fig. 2C, "yellow" needs to be corrected.
7. In Fig. 2D, do Chr07 and Chr15 represent the remaining genomic regions after excluding the SDR collinear regions? If yes, please clarify in the figure legend.
8. Pg14, Line 301, please clarify at which developmental stages these flower buds are.
9. Pg14, Line 300-305, how similar are the two intact RR genes?
10. Pg15, Line 310, "the first exon and upstream…a significantly higher methylation level…". It seems not to be the case in the first copy of the RR genes in S. arbutifolia.
11. Pg16, Line 347, "…showed higher similarity…" Higher than what?
12. Pg16-17, Line 348-353, "…originated before the divergence of the intact RR genes between willow and poplar." Why?

**Reviewer 2**

Repeated turnovers keep sex chromosomes young in willows

Overall comment:
Sex chromosome evolution in willows is fascinating. Wang et al. provides a nice contribution in understanding how sex chromosomes evolve in this group. I have some comments detailed below. The most major one is the need to better define what the authors consider the 'ever-young' hypothesis which usually refers to the 'foutain-of-youth' hypothesis which highlights the potential important role of limited recombination events in the maintenance of homomorphic sex chromosomes. However the authors seem to use 'ever-young' to refer to both the sex chromosome turnover and the 'foutain-of-youth' hypothesis. Clarification is needed.

Detailed comments:

l.38: "lack of obvious sex chromosome heteromorphism" - Unclear. Sex chromosome heteromorphism is often used to describe differences between the sex chromosomes at the karyotype level. Not sure what the authors mean by "obvious" heteromorphism. I would suggest deleting "obvious" or changing the term - maybe the authors mean "limited degeneration" instead?

L.46: What does "patterns" refer to here? Homo/hetero-morphy ? Length of non recombining region? Presence or not of rearrangements? Please describe more.

L.58 : Similar to my first comment. Heteromorphy and homomorphy describe sex chromosomes that are cytologically different or undistinguishable respectively. At the molecular level, the sex chromosome can differ in size though it would be limited (e.g. W/Y specific regions). Please add "at the karyotype level".

L.62: "sizes of homomorphic sex determination systems"? I think the authors mean "the size of the non-recombining regions" ? 

L.66 'ever-young' - The authors use this term from the cited paper "Ever-young sex chromosomes in European tree frogs." from Stöck et al. 2011. However, this paper shows evidence for the 'fountain-of-youth' hypothesis which posits that rare recombination events still occur between the sex chromosomes, maintaining their homomorphy. While the authors use the term to refer to both the sex chromosome turnover and rare recombination events which is confusing. The authors should delete " keeping them 'ever young' " and refer to the hypothesis involving rare recombination as "fountain-of-youth" (Perrin 2009).

Reference:
Perrin, N. (2009). Sex reversal: a fountain of youth for sex chromosomes?. Evolution: International Journal of Organic Evolution, 63(12), 3043-3049.


L.83: It would be nice to highlight that those hypotheses are hard to test and difficult to distinguish from the expectations under other hypotheses. I would recommend to cite the review from Palmer et al. 2019

Reference:
Palmer, D. H., Rogers, T. F., Dean, R., & Wright, A. E. (2019). How to identify sex chromosomes and their turnover. Molecular ecology, 28(21), 4709-4724.

L.88: what complexity means in this context, just diversity?

L.104 : pseudo-autosomal region? 

L.109-113: More explanation needed here. Others would argue that younger, smaller non-recombining regions might be more useful to investigate early stages of chromosome differentiation and the forces driving sex chromosome differentiation and their turnover; while in sex chromosome with large non recombining region, the drivers of sex chromosome turnover and evolution and the changes resulting from recombination cessation on the sex chromosome might be confounded.

L.124: Similar to my previous comment on 'ever' young hypothesis. It is not clear what the authors are referring to. If it refers to the 'fountain-of-youth' hypothesis, it would be better to use this term. In any case, it should be better defined.

L.183 : Any explanation about why those individuals had a higher level of heterozygosity? Needed to justify the exclusion of individuals.

For the results it might be useful to have a small summary table for the different SNP pattern containing information from both species together. It would help to simplify the results and the important information. Since the number of individuals is <= 30 for each sex of each species it would be nice to have the actual ratio (ex: 7/30 for 23.06% of females).

L.193-198: confusing

L.196: remove in depth 
L.199: "and are unable to align with female individuals (XX)," rewording needed - maybe something like this: "and thus no read from females (XX) map to these regions"
L.205-206: unclear
L.207-208: in all the females / all the males ?
L.228: more description needed for the type of SNPs (which would be nice in the suggested summary table). How many SNPs are heterozygous in all males and homozygous in all females? What are the other patterns observed here?
L.401: add "resulting in the emergence of a new sex chromosome" after "to an autosome"

**Authors’ response to reviewers**

Reviewer reports:
Reviewer #1: Evolution of sex chromosomes in the Salicaceae is particularly fascinating due to frequent sex chromosome turnover events. Wang et al. identified sex determination regions (SDRs) in Salix chaenomeloides and S. arbutifolia through genome-wide association study and performed comparative genomic analysis to trace the evolutionary history of sex chromosomes in Salix species. The manuscript addresses an interesting topic, and the methodological approaches are appropriate. Below are some suggestions for further improvement of the manuscript.
Reply: We appreciate the reviewer’s positive comments on our work. We have improved the original manuscript and addressed all of the concerns raised. We hope these modified contents will make the new manuscript more acceptable for publication.

1. Although the authors provided an accession number of the sequence data used in this study, the data is not released yet. All relevant data must be made available before the manuscript is accepted for publication.
Reply: Thanks for your careful checks. The data generated in this study has been uploaded to the database (https://bigd.big.ac.cn/bioproject) under accession number PRJCA005435 and will be released after the manuscript is accepted.

2. Pg 3, Line 49-50, please cite research articles, not review articles.
Reply: We have revised the references to cite the following research articles (Line 51):
Harkess A, Zhou J, Xu C, Bowers JE, Van der Hulst R, Ayyampalayam S, et al. The asparagus genome sheds light on the origin and evolution of a young Y chromosome. Nat Commun. 2017;8:1279.
Geraldes A, Hefer CA, Capron A, Kolosova N, Martinez‐Nuñez F, Soolanayakanahally RY, et al. Recent Y chromosome divergence despite ancient origin of dioecy in poplars (Populus). Mol Ecol. 2015;24:3243-3256.
Tennessen JA, Wei N, Straub SC, Govindarajulu R, Liston A, Ashman T-L. Repeated translocation of a gene cassette drives sex-chromosome turnover in strawberries. PLoS Biol. 2018;16:e2006062.

3. Please revise or make the correction for the sentences listed below.
Pg4, Line 69-72; Line 84-87
Reply: Corrected (Line 71-72; Line 89-92).

Pg6, Line 115-117; Line 121-122
Reply: Corrected (Line 123-126; Line 129-131).

Pg9-10, Line 197-201
Reply: Corrected (Line 205-210).

Pg10, Line 209-211
Reply: Corrected (Line 218-220).

Pg16, Line 338-341
Reply: Corrected (Line 348-350).

Pg21, Line 441, please replace "Whatever"
Reply: We have replaced "Whatever" with "In summary" (Line 448).

4. Pg5, Line 90-95, please include a reference listed below
Li, W., Wu, H., Li, X. et al. Fine mapping of the sex locus in Salix triandra confirms a consistent sex determination mechanism in genus Salix. Hortic Res 7, 64 (2020).
Li et al. also mapped the sex locus on LG XV in Salix triandra.
Reply: We have added the corresponding content and reference in the revised manuscript (Line 98).

5. In the figure legend of Fig. 1G, "red" needs to be corrected.
Reply: Corrected.

6. In the figure legend of Fig. 2C, "yellow" needs to be corrected.
Reply: Corrected.

7. In Fig. 2D, do Chr07 and Chr15 represent the remaining genomic regions after excluding the SDR collinear regions? If yes, please clarify in the figure legend.
Reply: Chr07 and chr15 represent all genomic regions of the chromosome. Chr07’ represents the autosomal genomic region in S. arbutifolia (which has the SDR on Chr15) that is collinear with the S. chaenomeloides SDR, and Chr15’ represents the autosomal genomic region in S. chaenomeloides (which has the SDR on Chr07) that is collinear with the S. arbutifolia SDR. We have improved these descriptions in the legend of Fig. 2D.

8. Pg14, Line 301, please clarify at which developmental stages these flower buds are.
Reply: The flower buds of S. chaenomeloides and S. arbutifolia were collected in September and October 2020, respectively. We have added this information in the methods section (Line 610).

9. Pg14, Line 300-305, how similar are the two intact RR genes?
Reply: The nucleotide sequence similarity of the two intact RR genes is greater than 98.6%. This result has been added at Line 311-312 in the revised manuscript.

10. Pg15, Line 310, "the first exon and upstream…a significantly higher methylation level…". It seems not to be the case in the first copy of the RR genes in S. arbutifolia.
Reply: Thanks for pointing this out. We agree with the reviewer and changed the description at Line 321 in the revised manuscript.

11. Pg16, Line 347, "…showed higher similarity…" Higher than what?
Reply: The 7 RR partial duplicates (SpuZ:S3-S9) of S. purpurea showed higher similarity with the RR partial duplicates of S. chaenomeloides and S. arbutifolia than the other two duplicates (SpuZ:S1-S2). We have added this information in the revised manuscript (Line 357).

12. Pg16-17, Line 348-353, "…originated before the divergence of the intact RR genes between willow and poplar." Why?
Reply: This conclusion was inferred from the relative positions of these RR partial duplicates in the phylogenetic relationship to the intact RR genes (Fig. 4B). Since the phylogenetic relationship showed that these RR partial duplicates clustered outside of the intact RR genes from willow and poplar, it was speculated that the origin of these partial duplicates might predate the divergence of the intact RR genes between willow and poplar.


Reviewer #2: Repeated turnovers keep sex chromosomes young in willows
Overall comment:
Sex chromosome evolution in willows is fascinating. Wang et al. provides a nice contribution in understanding how sex chromosomes evolve in this group. I have some comments detailed below. The most major one is the need to better define what the authors consider the 'ever-young' hypothesis which usually refers to the 'foutain-of-youth' hypothesis which highlights the potential important role of limited recombination events in the maintenance of homomorphic sex chromosomes. However the authors seem to use 'ever-young' to refer to both the sex chromosome turnover and the 'foutain-of-youth' hypothesis. Clarification is needed.
Reply: We appreciate the reviewer’s positive comments on our work. We apologize for the confusion caused by the use of "ever young" to refer to both the sex chromosome turnover and the "fountain of youth" hypothesis. Since our study focuses on sex chromosome turnover rather than rare recombination events, to avoid confusion, we have removed references to this term throughout the manuscript and rephrased sentences using the term as suggested by the reviewer.

Detailed comments:
1. L.38: "lack of obvious sex chromosome heteromorphism" - Unclear. Sex chromosome heteromorphism is often used to describe differences between the sex chromosomes at the karyotype level. Not sure what the authors mean by "obvious" heteromorphism. I would suggest deleting "obvious" or changing the term - maybe the authors mean "limited degeneration" instead?
Reply: Sorry for the confusion. We have changed the term to “limited degeneration” in the revised manuscript (Line 38).

2. L.46: What does "patterns" refer to here? Homo/hetero-morphy? Length of non recombining region? Presence or not of rearrangements? Please describe more.
Reply: Thanks for your suggestions. We have updated these descriptions with more detail in the revised manuscript (Line 46-49).

3. L.58 : Similar to my first comment. Heteromorphy and homomorphy describe sex chromosomes that are cytologically different or undistinguishable respectively. At the molecular level, the sex chromosome can differ in size though it would be limited (e.g. W/Y specific regions). Please add "at the karyotype level".
Reply: We have added “at the karyotype level” to the revised manuscript (Line 60).

4. L.62: "sizes of homomorphic sex determination systems"? I think the authors mean "the size of the non-recombining regions"?
Reply: Sorry for the inaccurate description, we have revised it according to the reviewer's suggestion (Line 64-65).

5. L.66 'ever-young' - The authors use this term from the cited paper "Ever-young sex chromosomes in European tree frogs." from Stöck et al. 2011. However, this paper shows evidence for the 'fountain-of-youth' hypothesis which posits that rare recombination events still occur between the sex chromosomes, maintaining their homomorphy. While the authors use the term to refer to both the sex chromosome turnover and rare recombination events which is confusing. The authors should delete " keeping them 'ever young' " and refer to the hypothesis involving rare recombination as "fountain-of-youth" (Perrin 2009).
Reference:
Perrin, N. (2009). Sex reversal: a fountain of youth for sex chromosomes?. Evolution: International Journal of Organic Evolution, 63(12), 3043-3049.
Reply: Sorry for the confusion caused by the use of the term 'ever young', we have deleted this term in the revised manuscript, referred to the hypothesis involving rare recombination as 'fountain-of-youth' and added the reference suggested by the reviewer (Line 66-69).

6. L.83: It would be nice to highlight that those hypotheses are hard to test and difficult to distinguish from the expectations under other hypotheses. I would recommend to cite the review from Palmer et al. 2019
Reference:
Palmer, D. H., Rogers, T. F., Dean, R., & Wright, A. E. (2019). How to identify sex chromosomes and their turnover. Molecular ecology, 28(21), 4709-4724.
Reply: As suggested by the reviewer, we have added the corresponding description and cited this reference in the revised manuscript (Line 76-88).

7. L.88: what complexity means in this context, just diversity?
Reply: The complexity here means the frequent turnover of sex determination systems in Salicaceae species (such as Müller et al. 2020; Yang et al. 2021), and the complex structure of sex chromosomes, such as palindromic structures of the S. purpurea SDR (Zhou et al. 2020) and evolutionary strata in S. viminalis (Almeida et al. 2020). In order to distinguish it from diversity, we changed it to ‘structural complexity’ (Line 93).
References:
Müller NA, Kersten B, Montalvão APL, Mähler N, Bernhardsson C, Bräutigam K, et al. A single gene underlies the dynamic evolution of poplar sex determination. Nat Plants. 2020;6:630-637.
Yang W, Wang D, Li Y, Zhang Z, Tong S, Li M, et al. A general model to explain repeated turnovers of sex determination in the Salicaceae. Mol Biol Evol. 2021;38:968-980.
Zhou R, Macaya-Sanz D, Carlson CH, Schmutz J, Jenkins JW, Kudrna D, et al. A willow sex chromosome reveals convergent evolution of complex palindromic repeats. Genome Biol. 2020;21:1-19.
Almeida P, Proux-Wera E, Churcher A, Soler L, Dainat J, Pucholt P, et al. Genome assembly of the basket willow, Salix viminalis, reveals earliest stages of sex chromosome expansion. BMC Biol. 2020;18:78.

8. L.104 : pseudo-autosomal region?
Reply: We have changed it to “autosomal or pseudo-autosomal region of chr19” (Line 109). Because the intact RR gene is located on the pseudo-autosomal region of chr19 in P. trichocarpa (Zhou et al. 2020) and P. tremula (Müller et al. 2020), and the autosomal region of chr19 in P. euphratica (Yang et al. 2021).
References:
Zhou R, Macaya-Sanz D, Schmutz J, Jenkins JW, Tuskan GA, DiFazio SP. Sequencing and analysis of the sex determination region of Populus trichocarpa. Genes. 2020;11:843.
Müller NA, Kersten B, Montalvão APL, Mähler N, Bernhardsson C, Bräutigam K, et al. A single gene underlies the dynamic evolution of poplar sex determination. Nat Plants. 2020;6:630-637.
Yang W, Wang D, Li Y, Zhang Z, Tong S, Li M, et al. A general model to explain repeated turnovers of sex determination in the Salicaceae. Mol Biol Evol. 2021;38:968-980.

9. L.109-113: More explanation needed here. Others would argue that younger, smaller non-recombining regions might be more useful to investigate early stages of chromosome differentiation and the forces driving sex chromosome differentiation and their turnover; while in sex chromosome with large non recombining region, the drivers of sex chromosome turnover and evolution and the changes resulting from recombination cessation on the sex chromosome might be confounded.
Reply: Thanks for the suggestion, there is indeed a possibility of an argument here. In general, the non-recombinant SDRs of willow are larger than that of poplar, possibly due to the partial overlap of willow SDRs with the centromere, selection for recombination suppression between the genes controlling sex determination and genes influencing the more obvious sexual dimorphism in willows, or differences in numbers of genes effecting sexual dimorphisms associated with pollination mode between insect-pollinated willows and wind-pollinated poplars. However, there is currently no evidence that poplars have younger sex chromosomes than willows, because they are shared across a wide taxonomic breadth including both Asian and North American species. In this case, the larger SDRs in willow contain more protein-coding genes and may provide more mutations to test the evolutionary forces driving sex chromosome turnover. These explanations have been added in the sections of background (Line 118-121) and discussion (Line 376-381) of the revised manuscript.

10. L.124: Similar to my previous comment on 'ever' young hypothesis. It is not clear what the authors are referring to. If it refers to the 'fountain-of-youth' hypothesis, it would be better to use this term. In any case, it should be better defined.
Reply: We have changed the statement in the revised manuscript (Line 132-133).

11. L.183: Any explanation about why those individuals had a higher level of heterozygosity? Needed to justify the exclusion of individuals.
Reply: We speculated that these three individuals may be recombinant individuals due to occasional recombination events between X- and Y-SDR. So these individuals have a higher level of heterozygosity. This phenomenon has also been found in other Salicaceae species (Geraldes et al. 2015; Sanderson et al. 2021). Therefore, we excluded them even if they did not influence the final conclusion (Line 192-194).
Reference:
Geraldes A, Hefer CA, Capron A, Kolosova N, Martinez‐Nuñez F, Soolanayakanahally RY, et al. Recent Y chromosome divergence despite ancient origin of dioecy in poplars (Populus). Mol Ecol. 2015;24:3243-3256.
Sanderson BJ, Feng G, Hu N, Carlson CH, Smart LB, Keefover-Ring K, et al. Sex determination through X-Y heterogamety in Salix nigra. Heredity. 2021;126:630-639.

12. For the results it might be useful to have a small summary table for the different SNP pattern containing information from both species together. It would help to simplify the results and the important information. Since the number of individuals is <= 30 for each sex of each species it would be nice to have the actual ratio (ex: 7/30 for 23.06% of females).
Reply: Thank you very much for this suggestion, we have added the summary tables for each species in the Additional file 2: Tables S5-S10.

13. L.193-198: confusing
Reply: We have updated the unclear description (Line 205-210).

14. L.196: remove in depth
Reply: Corrected (Line 205).

15. L.199: "and are unable to align with female individuals (XX)," rewording needed- maybe something like this: "and thus no read from females (XX) map to these regions"
Reply: Corrected (Line 208-209).

16. L.205-206: unclear
Reply: We have revised the unclear description (Line 213-217).

17. L.207-208: in all the females / all the males ?
Reply: We have updated this unclear description in the revised manuscript (Line 213-217).

18. L.228: more description needed for the type of SNPs (which would be nice in the suggested summary table). How many SNPs are heterozygous in all males and homozygous in all females? What are the other patterns observed here?
Reply: The summary table has been added in the Additional file 2: Table S9 as suggested by the reviewer.

19. L.401: add "resulting in the emergence of a new sex chromosome" after "to an autosome"
Reply: Added (Line 410-411).
